# Viperid Envenomation Wound Exudate Contributes to Increased Vascular Permeability via a DAMPs/TLR-4 Mediated Pathway

**DOI:** 10.3390/toxins8120349

**Published:** 2016-11-24

**Authors:** Alexandra Rucavado, Carolina A. Nicolau, Teresa Escalante, Junho Kim, Cristina Herrera, José María Gutiérrez, Jay W. Fox

**Affiliations:** 1 Instituto Clodomiro Picado, Facultad de Microbiología Universidad de Costa Rica, San José 11501-2060, Costa Rica; alexandra.rucavado@ucr.ac.cr (A.R.); teresa.escalante@ucr.ac.cr (T.E.); cristina.herreraarias@gmail.com (C.H.); 2 Laboratório de Toxinologia, Instituto Oswaldo Cruz, Rio de Janeiro CEP 21040-360, Brazil; carolnicolau.bio@gmail.com; 3 Department of Fine Chemistry & New Materials, Sangji University, Wonju-si, Kangwon-do 220-702, Korea; jhokim@sangji.ac.kr; 4 Facultad de Farmacia, Universidad de Costa Rica, San José 11501-2060, Costa Rica; 5 Department of Microbiology, Immunology and Cancer Biology, University of Virginia School of Medicine, P.O. Box 800734, Charlottesville, VA 22908, USA

**Keywords:** snake venom metalloproteinases (SVMPs), TLR4, damage associated molecular pattern molecules (DAMPs), exudate, increased vascular permeability

## Abstract

Viperid snakebite envenomation is characterized by inflammatory events including increase in vascular permeability. A copious exudate is generated in tissue injected with venom, whose proteomics analysis has provided insights into the mechanisms of venom-induced tissue damage. Hereby it is reported that wound exudate itself has the ability to induce increase in vascular permeability in the skin of mice. Proteomics analysis of exudate revealed the presence of cytokines and chemokines, together with abundant damage associated molecular pattern molecules (DAMPs) resulting from both proteolysis of extracellular matrix and cellular lysis. Moreover, significant differences in the amounts of cytokines/chemokines and DAMPs were detected between exudates collected 1 h and 24 h after envenomation, thus highlighting a complex temporal dynamic in the composition of exudate. Pretreatment of mice with Eritoran, an antagonist of Toll-like receptor 4 (TLR4), significantly reduced the exudate-induced increase in vascular permeability, thus suggesting that DAMPs might be acting through this receptor. It is hypothesized that an “Envenomation-induced DAMPs cycle of tissue damage” may be operating in viperid snakebite envenomation through which venom-induced tissue damage generates a variety of DAMPs which may further expand tissue alterations.

## 1. Introduction

Envenomation by viperid snakes causes local and systemic pathological and pathophysiological manifestations [1,2]. Many of these pathologies, such as hemorrhage and necrosis, have been associated with the action of the snake venom metalloproteinases (SVMPs) [3,4,5,6,7] which elicit these effects by both direct and indirect mechanisms [5,8]. One of the best described outcomes of the direct action of the SVMPs is hemorrhage, which is the result of proteolytic degradation of key extracellular matrix proteins in the host stroma and capillaries, allowing extravasation of capillary contents into the stroma [9,10,11,12]. In addition, viperid envenomation triggers an inflammatory response which likely contributes to these pathological features, as well as being involved in tissue repair and regeneration [13,14].

Several years ago we described in animal models of envenomation that there is a copious volume of wound exudate at the site of tissue damage [5,8]. Markers for disease and various pathological conditions are often best observed in proximity to the site of tissue damage. As such we utilized venom-induced wound exudate for proteomic exploration of the effects of whole venom and isolated toxins, as well as neutralization by antivenoms and toxin inhibitors, to gain insight into the biological mechanisms by which these agents in vivo give rise to the symptoms and pathologies observed during snakebite envenomation [15,16,17]. These studies, in addition to illuminating many aspects of toxin action, also demonstrated that venom-induced wound exudate is a very rich reservoir of various inflammatory mediators and of damage-associated (or danger-associated) molecular pattern molecules (DAMPs). Some of the DAMPs are derived from the proteolysis of host proteins by the SVMPs and activated endogenous host proteinases and some the result of cell lysis and the escape of cellular proteins into the exudate due to cytotoxic phopholipases A_2_ present in the venom [15].

DAMPs, like the pathogen-associated molecular pattern molecules (PAMPs), generate most of their biological activities via engagement of toll-like receptors (TLR) [18,19]. As their name implies DAMPs are the result of cellular and extracellular damage and serve to provide a host response to cellular injury by launching innate immunity inflammatory responses [20]. Prolonged or excessive DAMP response rather than being only beneficial to the host can in some situations also be deleterious. The microvascular endothelium is intimately related to microbial sepsis-induced organ failure by impacting the vascular barrier following engagement of endothelial TLRs by PAMPs [21,22]. Furthermore DAMP engagement with TLRs on endothelial surfaces has been shown to exacerbate the action of PAMPs-associated microbial sepsis [23]. In addition, DAMPs themselves have been implicated in ischemic disease, pulmonary disease, cancer and metastasis, ocular disease [24], and kidney injury [25]. Noteworthy many of these diseases share a fascinating level of overlapping pathophysiology with viperid envenomation, including increased vascular permeability. Thus, the possible role of DAMPs in the inflammatory scenario in snakebite envenomation requires exploration.

Previous works with snake venoms have shown a role for TLR2 as well as MYD88-dependent TLR signaling in venom-induced inflammation [26,27,28]. Those studies suggest a potential for increases in vascular permeability being mediated through these and other pathways. Given the critical role of increase in vascular permeability in envenomation pathophysiology we hypothesized that DAMPs and other envenomation-derived and inflammatory products found in wound exudate may play a functional role in envenomation. Here we report that wound exudate generated in a mouse model of viperid envenomation contain multiple cytokines and chemokines, as well as DAMPs, and induces increase in vascular permeability. Moreover, we show that this effect is likely to be mediated at least in part by TLR4, as demonstrated by its reduction by pretreatment with Eritoran, a specific TRL4 antagonist. These findings shed new light in the complex mechanisms involved in the inflammatory responses of tissue in snakebite envenomation and novel routes for therapeutic intervention to attenuate envenomation mortality and morbidity.

## 2. Results

### 2.1. Exudates Collected from Mice Injected with B. asper Venom Increase Vascular Permeability

**Figure 1:**
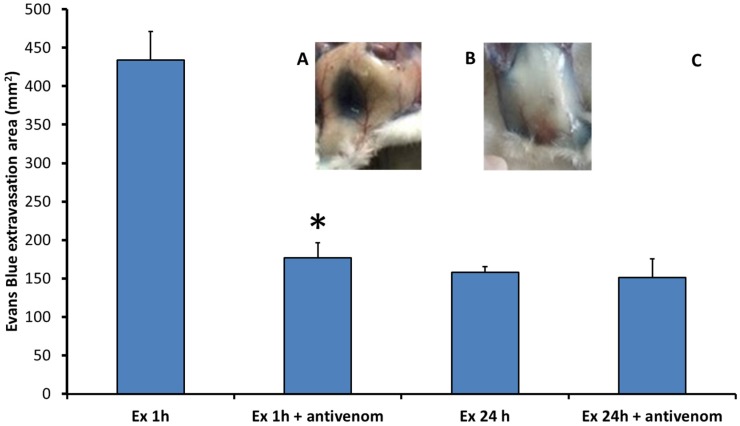
Wound exudate induces an increase in vascular permeability. Upper figures show samples of skin of mice injected intradermally with (**A**) wound exudate collected from mice 1 h after intramuscular injection of *B. asper* venom; or (**B**) blood plasma from untreated mice. Both groups of mice received an intravenous injection of Evans Blue solution before the injection of exudate or plasma (see Materials and Methods for details). Note the absence of Evans Blue extravasation in the control (B), whereas a clear extravasation was observed after injection of exudate (A); (**C**) Quantitative analysis of the increase in vascular permeability in mouse skin after injection of exudates collected 1 h and 24 h after injection of *B. asper* venom. In both types of exudates experiments were also performed with samples previously incubated with antivenom to neutralize the venom toxins present. One hour exudate induced a higher increase in vascular permeability than 24 h exudate. * A significant reduction in the activity by antivenom (*p* < 0.05) was observed only with 1 h exudate. Controls injected with mouse plasma alone or with antivenom alone did not show increase in vascular permeability.

When exudates collected from animals treated with *B. asper* venom were injected intradermally in the skin of mice, they induced an increase in vascular permeability, as reflected by the extravasation of Evans Blue (Figure 1). In order to assess whether this effect was due to the action of venom components present in the exudate, samples of exudate were incubated with polyvalent antivenom before testing in the mouse skin. As depicted in Figure 1, a large reduction in the effect was observed after incubation with antivenom in exudate of 1 h, but not in the neutralized exudate of 24 h, indicating that venom components play a role in the effect only in 1 h exudate samples. However, even in the 1 h exudate, there was a residual effect after neutralization by antivenom, indicating a venom-independent effect of exudate on permeability (Figure 1). As controls, normal mouse plasma and polyvalent antivenom did not induce an increase in vascular permeability (Figure 1). In order to attenuate concern that the observed effect was due to the presence of bacterial lipopolysaccharides, exudate collected at 24 h was incubated with polymyxin B before injection in mice. No reduction in the effect was observed, indicating that it is not due to the action of bacterial endotoxins. On the basis of these findings, the composition of the exudates in terms of inflammatory mediators was investigated.

### 2.2. Exudates Contain High Concentrations of Inflammatory Mediators

Given the clear capability of exudate to induce an increase in vascular permeability, it was necessary to investigate its molecular composition. When the cytokine and chemokine subproteome in exudates was analyzed by the Luminex technology, abundant inflammatory mediators were detected (Table 1). A dynamic development of the composition of the exudate was observed when comparing these subproteomes in exudates collected at 1h and 24 h, since a higher concentration of cytokines and chemokines was observed in the 24 h exudate as compared to the 1 h exudate. Among 32 mediators quantified, 14 of them presented more than 10-fold increase in concentration in the 24 h samples (Table 1). The highest increases were observed in IL-1β, CCL3, and CCL4. Thus, exudates, particularly the one collected at 24 h, contain abundant cytokines and chemokines.

### 2.3. Abundant DAMPs Are Identified in the Proteomes of Exudates

In order to ascertain whether exudates contained proteins that have been categorized as DAMPs, and which could play a role in the inflammatory event described, the full proteomes of exudates were analyzed (Table S1) vis-à-vis the information collected concerning the identity of DAMPs in exudates. As shown in Table 2, many proteins identified as DAMPs in the literature are observed present in both 1 h and 24 h exudates. When comparing the quantitative values of DAMPs in the exudates at the two time intervals, there is a clear trend towards higher abundance of many of these in the 24 h samples. Interesting exceptions are basement membrane-specific heparan sulfate proteoglycan core protein, 60 kDa heat shock protein (mitochondrial), and heat shock protein beta 2, whose quantitative values were higher in the 1 h exudate (Table 2). Thus, exudates contain a wide range of DAMPS, some of which are notably abundant at 24 h.

### 2.4. Eritoran, an Inhibitor of TLR4, Inhibits the Vascular Permeability Effect Induced by Exudate

**Figure 2:**
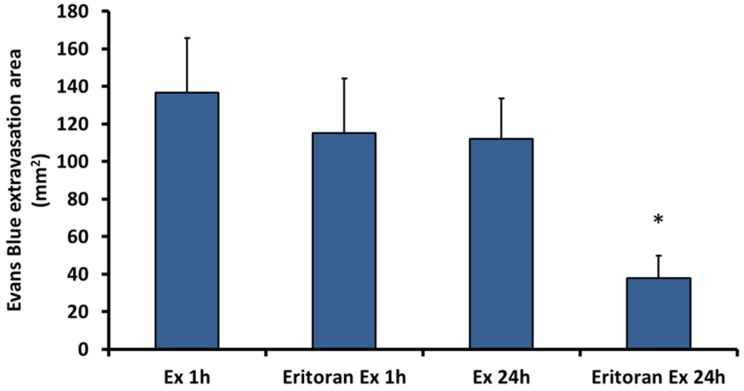
Effect of Eritoran in the increase of vascular permeability induced by exudates (Ex). Exudates were collected at 1 h and 24 h from mice injected with venom. Then, a separate group of mice were pretreated with either Eritoran or saline solution. Afterwards, these mice were injected intradermally in the skin with either 1 h exudate or 24 h exudate previously incubated with antivenom to neutralize venom toxins, as described in the legend of Figure 1. The increase in vascular permeability was assessed by extravasation of Evans blue, as described in Materials and Methods. The following experimental groups were used: Ex 1h: Mice injected with 1 h exudate; Eritoran Ex 1h: Mice pretreated with Eritoran and then injected with 1 h exudate; Ex 24h: Mice injected with 24 h exudate; Eritoran Ex 24h: Mice pretreated with Eritoran and then injected with 24 h exudate. Control mice pretreated with Eritoran and then injected intradermally with either mouse plasma or antivenom did not develop any extravasation of Evans blue. * Eritoran significantly reduced the effect induced by 24 h exudate (*p* < 0.05) but not by 1 h exudate.

The presence of abundant DAMPs in exudates prompted us to assess whether the effect of exudate in vascular permeability could be due to the action of DAMPs. Since many DAMPs act in the cells of the innate immune system through TLR4, the effect on vascular permeability after blocking this receptor with Eritoran was assessed. To this end, Eritoran was administered to mice before the injection of exudates collected from mice injected with venom. As shown in Figure 2, treatment with Eritoran significantly reduced the effect of exudates on vascular permeability, but only in the case of 24 h exudates. Thus, the increase in vascular permeability induced by 24 h exudate, but not by 1 h exudate, is mediated by TLR-4. We suggest this may be due to the presence of venom components in the 1 h exudate directly giving rise to permeability swamping out the TLR4 permeability axis and thus inhibition by Eritoran. Control mice receiving Eritoran and then injected intradermally with either normal mouse plasma or antivenom alone did not develop any extravasation of Evans blue in the skin.

## 3. Discussion

Snakebite envenomation involves highly complex and interrelated pathological and pathophysiological alterations which result from both the direct action of venom components on the host as well as a variety of tissue responses, in a dynamic interplay [7,29]. Many studies have assessed the direct action of venom toxins in the tissues, particularly of myotoxic PLA_2_s, serine proteinases, and hemorrhagic SVMPs [11,12,30]. Inflammation in venom-affected tissues is associated with edema, hyperalgesia, and recruitment of inflammatory cells. These effects are induced by a variety of mediators released in the tissues, including histamine, eicosanoids, nitric oxide, complement anaphylatoxins, bradykinin, and cytokines, among others [14,31]. The present investigation explores an aspect of this pathophysiology that to date has received little attention, i.e., the possible role of the exudate, generated in the tissue, as a reservoir of potent mediators in the inflammatory events characteristic of these envenomations.

The ability of exudates collected from venom-damaged tissue to induce increases in vascular permeability was used in our studies as an index of pro-inflammatory action of exudates. We deemed the increase in vascular permeability is one of the landmarks of envenomation-induced inflammation. Significantly, our results indicated that exudates collected at early and late time intervals after envenomation, i.e., 1 h and 24 h, induced an increase in vascular permeability when injected in the skin of mice. However, the effect induced by the 1 h exudate was significantly reduced when exudate was incubated with antivenom, whereas such inhibition did not occur in the case of the 24 h exudate. This indicates that the effect of 1 h exudate was predominantly due to residual venom components present in the tissues and in the exudate. In contrast, the effect of 24 h exudate does not seem to be caused by the direct action of venom toxins, but very likely due to tissue-derived and/or endogenously released inflammatory components. In agreement with our observations, it has been shown that *B. asper* venom concentration in the tissue, after an intramuscular injection in mice, is high at 1 h, being largely reduced by 24 h probably as a consequence of venom diffusion and systemic distribution [32].

In order to identify possible components in the exudate responsible for this inflammatory action, a subproteome analysis of cytokines and chemokines in exudates was performed. Our results revealed the presence of abundant inflammatory mediators. Interestingly, much higher abundance of these components was observed in the 24 h exudate, as compared to the 1 h exudate, indicating that the composition of the exudate varies in concordance with the extent of tissue damage and subsequent repair processes. This agrees with the variations observed in the overall proteomes of exudates at different time intervals in the same experimental model of envenomation by *B. asper* venom [33]. Hence, although an abundant volume of exudate is present in venom-affected tissue even at the early stages of envenomation, its composition varies significantly as a result of the complex dynamics of tissue damage and response to venom deleterious effects.

Exploring the composition of this complex inflammatory milieu in the form of the exudate proteome and function may provide clues for a deeper understanding of the tissue dynamics in snakebite envenomation. The observation that 24 h exudate has a higher concentration of cytokines and chemokines predicts that this exudate would be more active at stimulating inflammatory cells and processes. The high concentration of many of these mediators in the 24 h exudate is likely to depend, at least partially, on the abundant population of inflammatory cells in the damaged tissue at that time interval, as previously described [34]. These cells, in particular monocytes/macrophages, are known to synthesize many of these mediators [35,36]. These findings suggest that the recruitment of inflammatory cells to the site of tissue damage is associated with the synthesis of mediators which, in turn, recruit additional cells and, at the same time, stimulate these cells to produce more mediators and set up a cycle of expansion of the inflammatory response. This may explain the reparative and regenerative processes that follow the acute tissue damage, but also may exacerbate tissue damage, a delicate balance that needs to be further investigated.

In addition to cytokines and chemokines, the exudate generated in venom-damaged tissue is known to contain many proteins of various types derived from the affected cells and extracellular matrix [35]. We were particularly interested in the identification of DAMPs, which are endogenous molecules, or fragments of molecules, released in the tissues as a consequence of damage of cells and extracellular matrix [19,37,38]. After binding to pattern-recognition receptors in cells of the innate immune system, DAMPs stimulate the synthesis of inflammatory mediators and, therefore, participate in the overall response of tissues to cellular and extracellular damage [39,40]. DAMPs act in concert with chemokines to regulate the recruitment and trafficking of leukocytes in inflammation [35]. Since snake venoms induce drastic pathological events in tissues, it is likely that abundant DAMPs are generated upon venom injection. It has been shown that mitochondrial DNA, cytochrome C, and ATP are released in tissue affected by venoms of *Bothrops asper* and *Crotalus durissus* [41,42]. To further explore this phenomenon, we performed a complete proteomic analysis of exudates collected after injection of *B. asper* venom, and identified DAMPs in these exudates, on the basis of information available in the literature. As expected, many DAMPs were identified in exudates, strongly supporting the concept that venom-induced tissue damage results in the release of DAMPs, which add to the complexity of the local milieu that develops in snakebite envenomation.

The inflammatory effects exerted by many DAMPs are mediated through their recognition by TLR4, a pattern-recognition receptor present in the membrane a various types of inflammatory cells [43]. To assess whether exudate-induced vascular permeability might be mediated by DAMPs, we used Eritoran, a specific blocker of TLR4. Pretreatment of mice with Eritoran significantly reduced the increase in vascular permeability, thus supporting the view that signals mediated through TLR4, probably generated by DAMPs, contribute to the pro-inflammatory activity of 24 h exudate. TLR4 mediates the action of a number of DAMPs, such as hyaluronan fragments [44,45], S100A9 [46], heat shock protein 60 [47], soluble heparan sulfate [48], and fibronectin fragments [49]. Fibrinogen stimulates secretion of chemokines by macrophages through TLR4 [50]. Interestingly, TLR4 has been shown to exert a protective role in muscular tissue damage induced by the venom of *Bothrops jararacussu* [51]. Thus, TLR4 is likely to be a centerpiece in the detection of venom-induced tissue damage and in the stimulus to inflammation in this pathology. Previous studies have demonstrated the involvement of TLR2 in the modulation of the inflammatory response after injection of *Bothrops atrox* venom [28], and the participation of MyD88 adaptor protein in this inflammatory scenario. MyD88 is involved in cellular activation after binding of DAMPs to TLRs [26,52]. Thus, various pattern recognition receptors are likely to participate in the tissue responses in snakebite envenomation.

Among the DAMPs identified in the exudates collected from the tissue of mice injected with *B. asper* venom, several are known to play roles in inflammation. Examples are fibrinogen [15,17,33], which is known to stimulate chemokine secretion by macrophages through TLR4 [50]. In addition, fibrinogen products transmit activating signals to leukocytes through interactions with integrins, inducing cytokine secretion by these cells [53]. Fibronectin fragments, also identified in the exudates, are known to induce expression of matrix metalloproteinases (MMPs) [54], and fragments of extracellular matrix proteins, and of the glycosaminoglycan hyaluronic acid, as a consequence of hydrolysis by venom or tissue proteinases and hyaluronidases, exert a variety of pro-inflammatory roles [55,56]. Decorin, found in our proteomic analysis, stimulates the expression of TNF-α and IL-1β, and reduces the expression of the anti-inflammatory IL-10 [56]. Therefore, many of the DAMPs detected in exudates from venom-affected tissue are known to play a variety of pro-inflammatory roles and are probably involved in the tissue responses to venom toxins.

The relationship of various DAMPs with the observed increase in vascular permeability deserves consideration. Some DAMPs might increase vascular permeability indirectly, by stimulating inflammatory cells to synthesize cytokines or chemokines which in turn act on the microvasculature. However, some DAMPs may also directly interact with the endothelial cells in venules. This is the case of S100 proteins, which have been repeatedly detected in exudates collected from tissues affected by venom and toxins of *B. asper* [15,17,33]. S100 A8 and S100 A9 increase monolayer permeability in a human endothelial cell line [57]. Direct stimulation of endothelial cells by DAMPs is therefore likely to also contribute to an increase in vascular permeability, trafficking of inflammatory cells and additionally generate a procoagulant phenotype which might contribute to hemostatic alterations in envenomations [23]. Moreover, TLR4 is known to mediate the disruption of the vascular endothelial barrier in the lungs [58].

**Figure 3:**
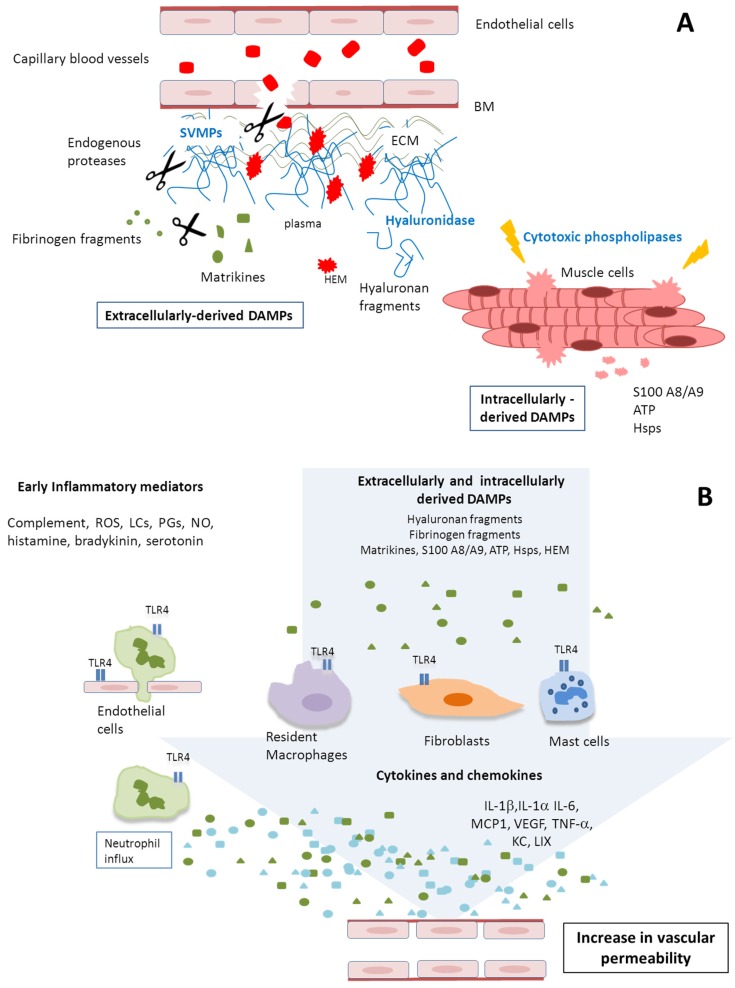
Hypothetical summary of the proposed events occurring in tissue injected with *B. asper* venom. (**A**) Venom toxins, particularly snake venom metalloproteinases (SVMPs), PLA_2_s and hyaluronidases, induce direct damage to the tissue, especially acute muscle fiber necrosis and degradation of extracellular matrix components, such as those of the basement membrane of capillary vessels, and other matrix molecules, including hyaluronic acid. Acute inflammation ensues, with the release of many types of mediators that promote an increase in vascular permeability, recruitment of inflammatory cells, and pain. Such acute tissue damage is also associated with the release of multiple damage associated molecular pattern molecules (DAMPs), both intracellular and extracellular; (**B**) DAMPs act on a variety of cells, including endothelial cells, other resident cells, and incoming inflammatory leucocytes, to generate diverse tissue responses, such as increase in vascular permeability, and the synthesis of a variety of cytokines and chemokines, which further contribute to the inflammatory scenario in a highly complex interplay. ROS: Reactive oxygen species; LCs: leukotrienes; PGs: prostaglandins; NO: Nitric oxide.

In conclusion, our results show that exudates collected from tissue damaged by *B. asper* snake venom toxins contain abundant cytokines and chemokines, as well as DAMPs, and is able to increase vascular permeability. In this context, early and late events take place in the tissue after venom injection, associated with the direct and indirect effects of venom components on muscle fibers and the microvasculature. This complex interplay of direct and indirect effects and early and late phenomena are hypothetically summarized in Figure 3. The key role of TLR4 in this phenomenon is centrally illustrated, suggesting that DAMP-mediated signals to inflammatory cells in the damaged tissue environment may be a significant and cyclic component in the overall inflammatory scenario giving rise to a “DAMPs derived tissue damage cycle” (DDTD cycle). As such we are further examining the role of DAMPs in snake venom-induced tissue damage in order to gain a more complete understanding of this complex pathological phenomenon, and to identify novel therapeutic avenues to attenuate envenomation mortality and morbidity.

## 4. Materials and Methods 

### 4.1. Venom

*B. asper* venom was obtained from more than 40 adult specimens collected in the Pacific region of Costa Rica and kept at the serpentarium of the Instituto Clodomiro Picado. After collection, venoms were pooled, lyophilized, and stored at −20 °C until used.

### 4.2. Exudate Collection

Groups of five CD-1 mice were injected intramuscularly (i.m.) with 50 μg *B. asper* venom, dissolved in 50 μL of apyrogenic 0.15 M NaCl (saline solution, SS). One and 24 h after venom injection, animals were sacrificed by CO_2_ inhalation, and an incision was made in the skin overlying the injected muscle, with care taken to avoid contamination. Wound exudate was collected from each animal individually into heparinized capillary tubes, pooled, and centrifuged to eliminate erythrocytes and other cells [15]. For some experiments, exudates were lyophilized and stored at −70° C until use. Exudates were reconstituted in the original volume with apyrogenic saline solution or water for further analyses. All experiments involving the use of mice were approved by the Institutional Committee for the Care and Use of Laboratory Animals (CICUA) of the University of Costa Rica (CICUA-025-15).

### 4.3. Increase in Vascular Permeability

Groups of 5 of CD-1 mice (18–20 g) received an intravenous (i.v.) injection of 200 μL of an Evans blue (EB; Sigma-Aldrich, St. Louis, MO, USA) solution (6 mg/mL; 60 mg/kg). Twenty min after EB injection, mice were injected intradermally (i.d.) with 50 μL of exudate samples collected 1 h and 24 h after venom injection in mice. In some groups, 40 μL of polyvalent antivenom (anti-*Bothrops*, *Crotalus* and *Lachesis* antivenom, Instituto Clodomiro Picado, Vázquez de Coronado, Costa Rica) was added to 250 μL of exudate and incubated for 20 min before injection, in order to neutralize the venom toxins that might be present in the exudates. Control groups of mice received either normal mouse plasma or antivenom. One hour after exudate injection or, in the case of controls, plasma or antivenom injection, animals were sacrificed, their skin was removed, and the areas of EB extravasation in the inner side of the skin were measured. To rule out the possibility that an increase in vascular permeability was due to the presence of bacterial lipopolysaccharide, exudate samples were incubated with polymyxin B (15 μg/mL) before testing in the mouse model as previously described [50].

### 4.4. Effect of Eritoran in Exudate-Induced Vascular Permeability

CD-1 mice (18–20 g) were injected i.m. with 50 µg *B. asper* venom, and exudate was collected 1 h and 24 h after envenomation, as described above. After that, two groups of 5 mice each were pretreated with either Eritoran (E5564; Eisai Co, Ltd., Woodcliff Lake, NJ, USA; 200 µg/100 µL i.v.) or SS (100 µL i.v.). After 1 h, mice received an intradermal injection of 50 µL of exudates collected at either 1 h or 24 h, previously incubated with antivenom to neutralize venom toxins, as described in Section 4.3. Control groups of mice were injected with Eritoran, as described, and then received an intradermal injection of 50 µL of either normal mouse plasma or antivenom. Increase in vascular permeability was assessed, as described above.

### 4.5. Quantification of Inflammatory Mediators in Exudates by Luminex Assays

For the analysis of the exudate subproteome associated with inflammatory mediators (i.e., cytokines and chemokines), 20 µL of each exudate (1 h or 24 h) were used for Luminex quantitative analysis of 32 analytes (Mouse Premixed Multi-Analyte Kit, R & D systems, Minneapolis, MN, USA) following the methodology recommended by the manufacturer.

### 4.6. Complete Proteomic Analysis of Wound Exudates and Identification of DAMPs

Lyophilized wound exudate samples were dissolved in water and protein quantification was performed using micro BCA protein assay kit (Thermo Scientific, Waltham, WA, USA). Twenty micrograms of protein from each sample was precipitated with acetone, resuspended in Laemmli buffer under reducing conditions and electrophoresed in a 5%–20% precast acrylamide gel (Bio-Rad, Hercules, CA, USA). The gel was stained with Coomassie Brilliant Blue and lanes were cut into 8 equal sized slices. Gel slices were destained for 3 h and the proteins in the gels were reduced (10 mM dithiothreitol, DTT) and alkylated (50 mM iodoacetamide) at room temperature. Gel slices were then washed with 100 mM ammonium bicarbonate, dehydrated with acetonitrile and dried in a speed vac, followed by in-gel digestion with a solution of Promega modified trypsin (20 ng/µL) in 50 mM ammonium bicarbonate for 30 min on ice. Excess trypsin solution was removed and the digestion continued for 18 h at 37 °C. The resulting tryptic peptides were extracted from gel slices with two 30 µL aliquots of a 50% acetonitrile/5% formic acid solution. These extracts were combined and evaporated to 15 µL for mass spectrometric (MS) analysis.

LC/MS/MS was performed using a Thermo Electron Orbitrap Velos ETD mass spectrometer system. Analytical columns were fabricated in-house by packing 0.5 cm of irregular C18 Beads (YMC Gel ODS-A, 12 nm, I-10-25 um) followed by 7.5 cm Jupiter 10 µm C18 packing material (Phenomenex, Torrance, CA, USA) into 360 × 75 µm fused silica (Polymicro Technologies, Phoenix, AZ, USA) behind a bottleneck. Samples were loaded directly onto these columns for the C18 analytical runs. 7 µL of the extract was injected, and the peptides were eluted from the column at 0.5 µL/min using an acetonitrile/0.1 M acetic acid gradient (2%–90% acetonitrile over 1 h). The instrument was set to Full MS (*m/z* 300–1600) resolution of 60,000 and programmed to acquire a cycle of one mass spectrum followed by collision-induced dissociation (CID) MS/MS performed in the ion trap on the twenty most abundant ions in a data-dependent mode. Dynamic exclusion was enabled with an exclusion list of 400 masses, duration of 60 s, and repeat count of 1. The electrospray voltage was set to 2.4 kV, and the capillary temperature was 265 °C.

The data were analyzed by database searching using the Sequest search algorithm in Proteome Discoverer 1.4.1 against the Uniprot Mouse database from July 2014. Spectra generated were searched using carbamidomethylation on cysteine as a fixed modification, oxidation of methionine as a variable modification, 10 ppm parent tolerance and 1 Da fragment tolerance. All hits were required to be fully tryptic. The results were exported to Scaffold (version 4.3.2, Proteome Software Inc., Portland, OR, USA) to validate MS/MS based peptide and protein identifications, and to visualize multiple datasets in a comprehensive manner. Proteins shown were identified in Scaffold with a confidence of 95%. The relative abundance of proteins was determined in Scaffold. This is a normalization algorithm that gives a unit less output of Quantitative Value as defined by the software provider, Proteome Software [59] based on averaging the unweighted spectral counts for all of the samples and then multiplying the spectrum counts in each sample by the average divided by the individual sample’s sum. The Quantitative Value allows a relative abundance comparison between a specific protein from different samples and relative abundance between proteins for a particular exudate sample.

DAMP proteins were identified in the exudate proteomic analysis, on the basis of the characterization of DAMPs in the literature. A list of DAMPs present in the proteomics analysis of exudate was prepared and the quantitative values of these proteins in 1 h and 24 h exudates were compared.

### 4.7. Statistical Analyses

The significance of the differences of mean values between experimental groups was assessed by analysis of variance (ANOVA), followed by Tukey-Kramer test to compare pairs of means. A *p* value < 0.05 was considered significant.

## Figures and Tables

**Table 1:** Cytokine profile (subproteome) of wound exudates collected at 1 h and 24 h.

Analytes (pg/mL)	Exudate 1 h	Exudate 24 h	Fold change *
CCL11 (EOTAXIN)	220.0	982.4	4.5
CSF-3 (G-CSF)	1670.0	>11,610.0	>6.9
CSF-2 (GM-CSF)	21.2	219.0	10.3
IFNy	3.2	37.8	11.8
IL-10	436.3	3419.0	7.8
IL-12p40	10.3	37.5	3.6
IL-12p70	5.2	23.8	4.6
IL-13	268.4	1217.0	4.5
IL-15	25.0	117.3	4.7
IL-17	<2.9	12.0	>4.1
IL-1a	228.3	5952.0	26.1
IL-1b	8.0	843.1	105.4
IL-2	4.8	9.2	1.9
IL-3	<2.4	10.2	>4.2
IL-4	<1.4	4.7	>1.9
IL-5	15.9	68.1	4.3
IL-6	7901.0	>17,536.0	>2.2
IL-7	3.8	10.5	2.8
IL-9	324.0	509.9	1.6
CXCL10 (IP-10)	52.6	2424.0	46.1
CXCL1/GRO alpha (KC)	4514.0	15,957.0	3.5
LIF	24.6	2252.0	91.5
CXCL5 (LIX)	1494.0	3817.0	2.5
CCL2 (MCP-1)	938.8	>18,874.0	>20.1
CSF-1 (M-CSF)	26.1	529.8	20.3
CXCL9 (MIG)	228.6	3034.0	13.3
CCL3 (MIP-1a)	27.9	>14,741.0	>528.3
CCL4 (MIP-1b)	80.7	>14,663.0	>181.7
CXCL2 (MIP-2)	4623.0	12,954.0	2.8
CCL5 (RANTES)	5.0	307.3	61.4
TNF-a	9.0	799.2	88.8
VEGF	<1.3	89.3	>68.7

Analyses were performed by using the Luminex quantitative analysis (see Materials and Methods for details). * Proteins showing a difference higher than 10-fold between exudates collected at the two times are highlighted.

**Table 2:** DAMPs identified in wound exudates collected 1 and 24 h after injection of *B. asper* venom.

Identified Proteins	Accession Number	Molecular Weight	Quantitative Value		Fold Change *
			1 h	24 h	
Hemoglobin subunit beta-2	P02089	16 kDa	745	1329	1.8
Fibronectin	P11276	273 kDa	274	290	1.0
Fibrinogen gamma chain	Q8VCM7	49 kDa	49	145	2.9
Heat shock cognate 71 kDa protein	P63017	71 kDa	50	17	2.9
Fibrinogen beta chain	Q8K0E8	55 kDa	12	107	8.9
Heat shock protein HSP 90-beta	P11499	83 kDa	41	26	1.6
Basement membrane-specific heparan sulfate proteoglycan core protein	B1B0C7	469 kDa	83	0	>83
Serum amyloid P-component	P12246	26 kDa	27	65	2.4
Histone H4	P62806	11 kDa	55	46	1.2
Histone H2B type 1-M	P10854	14 kDa	46	29	1.6
Proteoglycan 4	E9QQ17	111 kDa	18	12	1.5
Protein S100-A9	P31725	13 kDa	1	21	21
Myosin light chain 1/3, skeletal muscle isoform	P05977	21 kDa	10	59	5.9
Myosin-9	Q8VDD5	226 kDa	64	30	2.1
Serum amyloid A-4 protein	P31532	15 kDa	13	11	1.1
Myosin-10	Q3UH59	233 kDa	1	23	23
60 kDa heat shock protein	P63038	61 kDa	18	0	>18
40S ribosomal protein S19	Q9CZX8	16 kDa	0	34	>34
Decorin	P28654	40 kDa	1	22	22
Chondroitin sulfate proteoglycan 4	Q8VHY0	252 kDa	0	11	>11
Isoform 2 of Myosin-11	O08638-2	223 kDa	1	34	34
Myosin regulatory light chain 12B	Q3THE2	20 kDa	18	57	3.1
Endoplasmin	P08113	92 kDa	1	45	45
Heat shock protein beta-1	P14602	23 kDa	37	45	1.2
Calreticulin	P14211	48 kDa	0	22	>22
Protein S100-A8	P27005	10 kDa	0	80	>80
Isoform Smooth muscle of Myosin	Q60605-2	17 kDa	1	12	12
Myosin light chain 3	P09542	22 kDa	0	14	>14
Heat shock protein beta-2	Q99PR8	20 kDa	27	0	>27
Biglycan	P28653	42 kDa	0	22	>22
Serum amyloid A-1 protein	P05366	14 kDa	0	16	>16

* Proteins showing a difference higher than 10-fold between exudates collected at the two times are highlighted.

## Supplementary Materials

The following are available online at www.mdpi.com/2072-6651/8/12/349/s1, Table S1: List of all proteins identified by proteomics analysis in exudates collected at 1 h and 24 h after injection of *B. asper* venom. Quantitative values for all proteins are included.

## References

[1] Warrell D.A., Campbell J.A., Lamar W.W. (2004). Snakebites in Central and South America: Epidemiology, clinical features, and clinical management. The Venomous Reptiles of the Western Hemisphere.

[2] Warrell D.A. (2010). Snake bite. Lancet.

[3] Fox J.W., Bjarnason J.B. (1983). New proteases from *Crotalus atrox* venom. J. Toxicol. Toxin Rev..

[4] Bjarnason J.B., Fox J.W. (1988). Hemorrhagic toxins from snake venoms. J. Toxicol. Toxin Rev..

[5] Gutiérrez J.M., Rucavado A. (2000). Snake venom metalloproteinases: Their role in the pathogenesis of local tissue damage. Biochimie.

[6] Fox J.W., Serrano S.M.T. (2005). Structural considerations of the snake venom metalloproteinases, key members of the M12 reprolysin family of metalloproteinases. Toxicon.

[7] Gutiérrez J.M., Rucavado A., Escalante T., Lomonte B., Angulo Y., Fox J.W. (2010). Tissue pathology induced by snake venoms: How to understand a complex pattern of alterations from a systems biology perspective?. Toxicon.

[8] Gallagher P.G., Bao Y., Serrano S.M.T., Kamiguti A.S., Theakston R.D.G., Fox J.W. (2003). Use of microarrays for investigating the subtoxic effects of snake venoms: Insights into venom-induced apoptosis in human umbilical vein endothelial cells. Toxicon.

[9] Baramova E.N., Shannon J.D., Bjarnason J.B., Fox J.W. (1990). Identification of the cleavage sites by a hemorrhagic metalloproteinase in type IV collagen. Matrix.

[10] Baramova E.N., Shannon J.D., Fox J.W., Bjarnason J.B. (1991). Proteolytic digestion of non-collagenous basement membrane proteins by the hemorrhagic metalloproteinase Ht-e from *Crotalus atrox* venom. Biomed. Biochim. Acta.

[11] Gutiérrez J.M., Rucavado A., Escalante T., Díaz C. (2005). Hemorrhage induced by snake venom metalloproteinases: Biochemical and biophysical mechanisms involved in microvessel damage. Toxicon.

[12] Escalante T., Ortiz N., Rucavado A., Sanchez E.F., Richardson M., Fox J.W., Gutiérrez J.M. (2011). Role of collagens and perlecan in microvascular stability: Exploring the mechanism of capillary vessel damage by snake venom metalloproteinases. PLoS ONE.

[13] Gallagher P., Bao Y., Serrano S.M.T., Laing G.D., Theakston R.D.G., Gutiérrez J.M., Escalante T., Zigrino P., Moura-Da-Silva A.M., Nischt R. (2005). Role of the snake venom toxin jararhagin in proinflammatory pathogenesis: In vitro and in vivo gene expression analysis of the effects of the toxin. Arch. Biochem. Biophys..

[14] Teixeira C., Cury Y., Moreira V., Picolo G., Chaves F. (2009). Inflammation induced by *Bothrops asper* venom. Toxicon.

[15] Escalante T., Rucavado A., Pinto A.F.M., Terra R.M.S., Gutiérrez J.M., Fox J.W. (2009). Wound exudate as a proteomic window to reveal different mechanisms of tissue damage by snake venom toxins. J. Proteome Res..

[16] Rucavado A., Escalante T., Shannon J.D., Ayala-Castro C.N., Villalta M., Gutiérrez J.M., Fox J.W. (2012). Efficacy of IgG and F(ab′) 2 antivenoms to neutralize snake venom-induced local tissue damage as assessed by the proteomic analysis of wound exudate. J. Proteome Res..

[17] Rucavado A., Escalante T., Shannon J., Gutiérrez J.M., Fox J.W. (2011). Proteomics of wound exudate in snake venom-induced pathology: Search for biomarkers to assess tissue damage and therapeutic success. J. Proteome Res..

[18] Newton K., Dixit V.M. (2012). Signaling in innate immunity and inflammation. Cold Spring Harb. Perspect. Biol..

[19] Piccinini A.M., Midwood K.S. (2010). DAMPening inflammation by modulating TLR signalling. Mediat. Inflamm..

[20] Seong S.-Y., Matzinger P. (2004). Hydrophobicity: An ancient damage-associated molecular pattern that initiates innate immune responses. Nat. Rev. Immunol..

[21] Aird W.C. (2003). The role of the endothelium in severe sepsis and multiple organ dysfunction syndrome. Blood.

[22] Kumar P., Shen Q., Pivetti C.D., Lee E.S., Wu M.H., Yuan S.Y. (2009). Molecular mechanisms of endothelial hyperpermeability: Implications in inflammation. Expert Rev. Mol. Med..

[23] Khakpour S., Wilhelmsen K., Hellman J. (2015). Vascular endothelial cell Toll-like receptor pathways in sepsis. Innate Immun..

[24] Park-Windhol C., D’Amore P.A. (2016). Disorders of vascular permeability. Annu. Rev. Pathol. Mech. Dis..

[25] Allam R., Scherbaum C.R., Darisipudi M.N., Mulay S.R., Hägele H., Lichtnekert J., Hagemann J.H., Rupanagudi K.V., Ryu M., Schwarzenberger C. (2012). Histones from dying renal cells aggravate kidney injury via TLR2 and TLR4. J. Am. Soc. Nephrol..

[26] Moreira V., Teixeira C., Borges da Silva H., D’Império Lima M.R., Dos-Santos M.C. (2013). The crucial role of the MyD88 adaptor protein in the inflammatory response induced by *Bothrops atrox* venom. Toxicon.

[27] Giannotti K.C., Leiguez E., Moreira V., Nascimento N.G., Lomonte B., Gutiérrez J.M., Lopes de Melo R., Teixeira C. (2013). A Lys49 phospholipase A2, isolated from *Bothrops asper* snake venom, induces lipid droplet formation in macrophages which depends on distinct signaling pathways and the *C*-terminal region. Biomed. Res. Int..

[28] Moreira V., Teixeira C., Borges da Silva H., D’Império Lima M.R., Dos-Santos M.C. (2016). The role of TLR2 in the acute inflammatory response induced by *Bothrops atrox* snake venom. Toxicon.

[29] Gutiérrez J.M., Rucavado A., Chaves F., Díaz C., Escalante T. (2009). Experimental pathology of local tissue damage induced by *Bothrops asper* snake venom. Toxicon.

[30] Gutiérrez J.M., Ownby C.L. (2003). Skeletal muscle degeneration induced by venom phospholipases A 2: Insights into the mechanisms of local and systemic myotoxicity. Toxicon.

[31] Teixeira C.F.P., Zamunér S.R., Zuliani J.P., Fernandes C.M., Cruz-Hofling M.A., Fernandes I., Chaves F., Gutiérrez J.M. (2003). Neutrophils do not contribute to local tissue damage, but play a key role in skeletal muscle regeneration, in mice injected with *Bothrops asper* snake venom. Muscle Nerve.

[32] Saravia-Otten P., Robledo B., Escalante T., Bonilla L., Rucavado A., Lomonte B., Hernández R., Flock J.I., Gutiérrez J.M., Gastaldello S. (2013). Homogenates of skeletal muscle injected with snake venom inhibit myogenic differentiation in cell culture. Muscle Nerve.

[33] Herrera C., Macêdo J.K.A., Feoli A., Escalante T., Rucavado A., Gutiérrez J.M., Fox J.W. (2016). Muscle tissue damage induced by the venom of *Bothrops asper*: Identification of early and late pathological events through proteomic analysis. PLoS Negl. Trop. Dis..

[34] Gutiérrez J.M., Chaves F., Cerdas L. (1986). Inflammatory infiltrate in skeletal muscle injected with *Bothrops asper* venom. Rev. Biol. Trop..

[35] Mahdavian Delavary B., van der Veer W.M., van Egmond M., Niessen F.B., Beelen R.H.J. (2011). Macrophages in skin injury and repair. Immunobiology.

[36] Brancato S.K., Albina J.E. (2011). Wound macrophages as key regulators of repair: Origin, phenotype, and function. Am. J. Pathol..

[37] Schaefer L. (2014). Complexity of danger: The diverse nature of damage-associated molecular patterns. J. Biol. Chem..

[38] Vénéreau E., Ceriotti C., Bianchi M.E. (2015). DAMPs from cell death to new life. Front. Immunol..

[39] Yang D., Wei F., Tewary P., Howard O.M.Z., Oppenheim J.J. (2013). Alarmin-induced cell migration. Eur. J. Immunol..

[40] Turner N.A. (2016). Inflammatory and fibrotic responses of cardiac fibroblasts to myocardial damage associated molecular patterns (DAMPs). J. Mol. Cell. Cardiol..

[41] Zornetta I., Caccin P., Fernandez J., Lomonte B., Gutierrez J.M., Montecucco C. (2012). Envenomations by *Bothrops* and *Crotalus* snakes induce the release of mitochondrial alarmins. PLoS Negl. Trop. Dis..

[42] Cintra-Francischinelli M., Caccin P., Chiavegato A., Pizzo P., Carmignoto G., Angulo Y., Lomonte B., Gutiérrez J.M., Montecucco C. (2010). *Bothrops* snake myotoxins induce a large efflux of ATP and potassium with spreading of cell damage and pain. Proc. Natl. Acad. Sci. USA.

[43] Deguchi A., Tomita T., Ohto U., Takemura K., Kitao A., Akashi-Takamura S., Miyake K., Maru Y. (2016). Eritoran inhibits S100A8-mediated TLR4/MD-2 activation and tumor growth by changing the immune microenvironment. Oncogene.

[44] Taylor K.R., Trowbridge J.M., Rudisill J.A., Termeer C.C., Simon J.C., Gallo R.L. (2004). Hyaluronan fragments stimulate endothelial recognition of injury through TLR4. J. Biol. Chem..

[45] Voelcker V., Gebhardt C., Averbeck M., Saalbach A., Wolf V., Weih F., Sleeman J., Anderegg U., Simon J. (2008). Hyaluronan fragments induce cytokine and metalloprotease upregulation in human melanoma cells in part by signalling via TLR4. Exp. Dermatol..

[46] Tsai S.-Y., Segovia J.A., Chang T.-H., Morris I.R., Berton M.T., Tessier P.A., Tardif M.R., Cesaro A., Bose S. (2014). DAMP molecule S100A9 acts as a molecular pattern to enhance inflammation during influenza A virus infection: Role of DDX21-TRIF-TLR4-MyD88 pathway. PLoS Pathog..

[47] Ohashi K., Burkart V., Flohé S., Kolb H. (2000). Cutting edge: Heat shock protein 60 is a putative endogenous ligand of the toll-like receptor-4 complex. J. Immunol..

[48] Johnson G.B., Brunn G.J., Kodaira Y., Platt J.L. (2002). Receptor-mediated monitoring of tissue well-being via detection of soluble heparan sulfate by Toll-like receptor 4. J. Immunol..

[49] Okamura Y., Watari M., Jerud E.S., Young D.W., Ishizaka S.T., Rose J., Chow J.C., Strauss J.F. (2001). The extra domain A of fibronectin activates Toll-like receptor 4. J. Biol. Chem..

[50] Smiley S.T., King J.A., Hancock W.W. (2001). Fibrinogen stimulates macrophage chemokine secretion through toll-like receptor 4. J. Immunol..

[51] Paiva-Oliveira E.L., Ferreira da Silva R., Correa Leite P.E., Cogo J.C., Quirico-Santos T., Lagrota-Candido J. (2012). TLR4 signaling protects from excessive muscular damage induced by Bothrops jararacussu snake venom. Toxicon.

[52] Leiguez E., Giannotti K.C., Moreira V., Matsubara M.H., Gutiérrez J.M., Lomonte B., Rodríguez J.P., Balsinde J., Teixeira C. (2014). Critical role of TLR2 and MyD88 for functional response of macrophages to a group IIA-secreted phospholipase A2 from snake venom. PLoS ONE.

[53] Davalos D., Akassoglou K. (2012). Fibrinogen as a key regulator of inflammation in disease. Semin. Immunopathol..

[54] Saito S., Yamaji N., Yasunaga K., Saito T., Matsumoto S., Katoh M., Kobayashi S., Masuho Y. (1999). The fibronectin extra domain A activates matrix metalloproteinase gene expression by an interleukin-1-dependent mechanism. J. Biol. Chem..

[55] Kelsh R.M., McKeown-Longo P.J. (2013). Topographical changes in extracellular matrix: Activation of TLR4 signaling and solid tumor progression. Trends Cancer Res..

[56] Järveläinen H., Sainio A., Wight T.N. (2015). Pivotal role for decorin in angiogenesis. Matrix Biol..

[57] Wang L., Luo H., Chen X., Jiang Y., Huang Q. (2014). Functional characterization of S100A8 and S100A9 in altering monolayer permeability of human umbilical endothelial cells. PLoS ONE.

[58] Tauseef M., Knezevic N., Chava K.R., Smith M., Sukriti S., Gianaris N., Obukhov A.G., Vogel S.M., Schraufnagel D.E., Dietrich A. (2012). TLR4 activation of TRPC6-dependent calcium signaling mediates endotoxin-induced lung vascular permeability and inflammation. J. Exp. Med..

[59] Proteome Software. http://www.proteomesoftware.com.

