# Supplementary material for: Viperid Envenomation Wound Exudate Contributes to Increased Vascular Permeability via a DAMPs/TLR-4 Mediated Pathway
Source: Toxins (Basel). 2016 Nov 24;8(12):349. doi: 10.3390/toxins8120349 (PMC5198544; doi:10.3390/toxins8120349)
Supplement: Supplementary file 1 [file toxins-08-00349-s001.pdf]

Supplementary Materials: Viperid Envenomation Wound Exudate Contributes to Increased Vascular Permeability via a DAMPs/TLR-4 Mediated Pathway

Alexandra Rucavado, Carolina A. Nicolau, Teresa Escalante, Junho Kim, Cristina Herrera, José María Gutiérrez and Jay W. Fox

Table S1. List of all proteins identified by proteomics analysis in exudates collected at 1 h and 24 h after injection of *B. asper* venom. Quantitative values for all proteins are included.

Identified Proteins	Accession Number	Molecular Weight	Quantitative Value	
			1 h	24 h
Serum albumin OS = Mus musculus GN = Alb PE = 1 SV = 3	P07724	69 kDa	3152.60	3399.20
Cluster of Serotransferrin OS = Mus musculus GN = Tf PE = 1 SV = 1 (Q921I1)	Q921I1 [3]	77 kDa	1190.50	1228.80
Cluster of Isoform 3 of Titin OS = Mus musculus GN = Ttn (A2ASS6-3)	A2ASS6-3 [6]	619 kDa	629.96	228.82
Cluster of Hemoglobin subunit beta-2 OS = Mus musculus GN = Hbb-b2 PE = 1 SV = 2 (P02089)	P02089 [3]	16 kDa	745.6	1329.50
Cluster of Complement C3 OS = Mus musculus GN = C3 PE = 1 SV = 3 (P01027)	P01027	186 kDa	691.94	883.26
Cluster of Serine protease inhibitor A3K OS = Mus musculus GN = Serpina3k PE = 1 SV = 2 (P07759)	P07759 [4]	47 kDa	821.45	1291.70
Cluster of Alpha-2-macroglobulin OS = Mus musculus GN = Pzp PE = 4 SV = 1 (D3YW52)	D3YW52 [2]	167 kDa	781.67	711.64
Cluster of Alpha-1-antitrypsin 1-2 OS = Mus musculus GN = Serpina1b PE = 1 SV = 2 (P22599)	P22599 [4]	46 kDa	460.68	694.48
Creatine kinase M-type OS = Mus musculus GN = Ckm PE = 1 SV = 1	P07310	43 kDa	651.24	96.106
Cluster of Myosin-4 OS = Mus musculus GN = Myh4 PE = 1 SV = 1 (Q5SX39)	Q5SX39 [9]	223 kDa	43.478	528.58
Apolipoprotein A-I OS = Mus musculus GN = Apoa1 PE = 1 SV = 2	Q00623	31 kDa	292.32	183.06
Hemopexin OS = Mus musculus GN = Hpx PE = 1 SV = 2	Q91X72	51 kDa	264.57	355.82
Cluster of Alpha globin 1 OS = Mus musculus GN = haemaglobin alpha 1 PE = 1 SV = 1 (Q91VB8)	Q91VB8	15 kDa	233.11	435.91
Cluster of Phosphorylase OS = Mus musculus GN = Pygm PE = 1 SV = 1 (E9PUM3)	E9PUM3 [3]	88 kDa	299.72	46.909
Cluster of Fructose-bisphosphate aldolase OS = Mus musculus GN = Aldoa PE = 2 SV = 1 (A6ZI44)	A6ZI44 [3]	45 kDa	357.07	108.69
Murinoglobulin-1 OS = Mus musculus GN = Mug1 PE = 1 SV = 3	P28665	165 kDa	523.58	432.48
Fibronectin OS = Mus musculus GN = Fn1 PE = 1 SV = 4	P11276	273 kDa	274.74	290.61
Cluster of Ceruloplasmin OS = Mus musculus GN = Cp PE = 4 SV = 1 (G3X8Q5)	G3X8Q5 [2]	124 kDa	210.91	258.57
Cluster of Actin, alpha skeletal muscle OS = Mus musculus GN = Acta1 PE = 1 SV = 1 (P68134)	P68134 [7]	42 kDa	120.26	219.67
Cluster of Beta-enolase OS = Mus musculus GN = Eno3 PE = 1 SV = 3 (P21550)	P21550 [3]	47 kDa	215.54	73.223
Cluster of Complement factor H OS = Mus musculus GN = Cfh PE = 1 SV = 1 (E9Q8I0)	E9Q8I0	141 kDa	160.03	148.73
Cluster of Alpha-actinin-2 OS = Mus musculus GN = Actn2 PE = 1 SV = 2 (Q9JI91)	Q9JI91 [3]	104 kDa	189.64	59.494
Apolipoprotein A-IV OS = Mus musculus GN = Apoa4 PE = 1 SV = 3	P06728	45 kDa	120.26	216.24
Apolipoprotein B-100 OS = Mus musculus GN = Apob PE = 1 SV = 1	E9Q414	509 kDa	144.31	144.16
Vitamin D-binding protein OS = Mus musculus GN = Gc PE = 1 SV = 2	P21614	54 kDa	177.61	133.86
Cluster of Isoform M1 of Pyruvate kinase PKM OS = Mus musculus GN = Pkm (P52480-2)	P52480-2 [2]	58 kDa	195.19	81.232
Plasminogen OS = Mus musculus GN = Plg PE = 1 SV = 3	P20918	91 kDa	142.46	107.55

Table S1. *Cont.*

Identified Proteins	Accession Number	Molecular Weight	Quantitative Value	
			1 h	24 h
Cluster of Keratin, type II cytoskeletal 2 epidermal OS = Mus musculus GN = Krt2 PE = 1 SV = 1 (Q3TTY5)	Q3TTY5 [9]	71 kDa	94.356	90.385
Transthyretin OS = Mus musculus GN = Ttr PE = 1 SV = 1	P07309	16 kDa	144.31	127
Complement C4-B OS = Mus musculus GN = C4b PE = 1 SV = 3	P01029	193 kDa	134.13	108.69
Cluster of Inter alpha-trypsin inhibitor, heavy chain 4 OS = Mus musculus GN = Itih4 PE = 1 SV = 2 (A6X935)	A6X935 [2]	105 kDa	100.83	300.9
Cluster of Carboxylesterase 1C OS = Mus musculus GN = Ces1c PE = 1 SV = 4 (P23953)	P23953 [3]	61 kDa	199.81	144.16
Alpha-actinin-3 OS = Mus musculus GN = Actn3 PE = 2 SV = 1	O88990	103 kDa	160.96	54.917
Cluster of Uncharacterized protein OS = Mus musculus GN = Gm12117 PE = 3 SV = 1 (V9GXK0)	V9GXK0 [3]	16 kDa	273.82	36.612
Cluster of Apolipoprotein E OS = Mus musculus GN = Apoe PE = 1 SV = 2 (P08226)	P08226	36 kDa	123.96	132.72
Protein Fga OS = Mus musculus GN = Fga PE = 4 SV = 1	E9PV24	87 kDa	33.302	179.63
Prolow-density lipoprotein receptor-related protein 1 OS = Mus musculus GN = Lrp1 PE = 1 SV = 1	Q91ZX7	505 kDa	0	45.765
Cluster of Filamin-C OS = Mus musculus GN = Flnc PE = 1 SV = 3 (Q8VHX6)	Q8VHX6 [4]	291 kDa	111.01	17.162
Fibrinogen gamma chain OS = Mus musculus GN = Fgg PE = 2 SV = 1	Q8VCM7	49 kDa	49.953	145.3
Fatty acid synthase OS = Mus musculus GN = Fasn PE = 1 SV = 2	P19096	272 kDa	84.18	32.035
Cluster of Ig heavy chain Mem5 (Fragment) OS = Mus musculus PE = 1 SV = 1 (P84751)	P84751	25 kDa	79.555	127
Gelsolin OS = Mus musculus GN = Gsn PE = 1 SV = 3	P13020	86 kDa	75.855	91.529
Cluster of Protein Gm20547 OS = Mus musculus GN = Gm20547 PE = 3 SV = 1 (B8JJN0)	B8JJN0 [3]	142 kDa	72.154	73.223
Cluster of L-lactate dehydrogenase OS = Mus musculus GN = Ldha PE = 1 SV = 1 (G5E8N5)	G5E8N5 [2]	40 kDa	104.53	34.323
Myosin-binding protein C, fast-type OS = Mus musculus GN = Mybpc2 PE = 1 SV = 1	Q5XKE0	127 kDa	121.18	45.765
Cluster of Isoform 3 of Keratin, type I cytoskeletal 10 OS = Mus musculus GN = Krt10 (P02535-3)	P02535-3 [5]	50 kDa	79.555	62.926
Isoform 2 of Ig gamma-2B chain C region OS = Mus musculus GN = Igh-3	P01867-2	37 kDa	74.005	80.088
Antithrombin-III OS = Mus musculus GN = Serpinc1 PE = 1 SV = 1	P32261	52 kDa	54.578	57.206
Cluster of Kininogen-1 OS = Mus musculus GN = Kng1 PE = 1 SV = 1 (O08677)	O08677 [2]	73 kDa	116.56	75.512
Cluster of Sarcoplasmic/endoplasmic reticulum calcium ATPase 1 OS = Mus musculus GN = Atp2a1 PE = 2 SV = 1 (Q8R429)	Q8R429 [2]	109 kDa	80.48	0
Alpha-2-HS-glycoprotein OS = Mus musculus GN = Ahsg PE = 1 SV = 1	P29699	37 kDa	47.178	80.088
Murinoglobulin-2 OS = Mus musculus GN = Mug2 PE = 2 SV = 2	P28666	162 kDa	273.82	183.06
Cluster of Protein Tnxb OS = Mus musculus GN = Tnxb PE = 4 SV = 1 (O35452)	O35452 [2]	435 kDa	32.377	22.882
Triosephosphate isomerase OS = Mus musculus GN = Tpi1 PE = 1 SV = 4	P17751	32 kDa	98.056	27.459
Cluster of Fibrillin-1 OS = Mus musculus GN = Fbn1 PE = 4 SV = 1 (A2AQ53)	A2AQ53 [2]	312 kDa	0	0
Cluster of Phosphoglycerate kinase 1 OS = Mus musculus GN = Pgk1 PE = 1 SV = 4 (P09411)	P09411	45 kDa	58.279	14.873
Haptoglobin OS = Mus musculus GN = Hp PE = 1 SV = 1	Q61646	39 kDa	60.129	83.52
Carbonic anhydrase 3 OS = Mus musculus GN = Ca3 PE = 1 SV = 3	P16015	29 kDa	55.503	25.171
Inter-alpha trypsin inhibitor, heavy chain 2 OS = Mus musculus GN = Itih2 PE = 1 SV = 1	G3X977 (+1)	106 kDa	32.377	57.206
Ig gamma-1 chain C region, membrane-bound form OS = Mus musculus GN = Ighg1 PE = 1 SV = 2	P01869	43 kDa	73.08	57.206
Cluster of Inhibitor of carbonic anhydrase OS = Mus musculus GN = Ica PE = 1 SV = 1 (Q9DBD0)	Q9DBD0	77 kDa	58.279	61.782
Phosphoglycerate mutase 2 OS = Mus musculus GN = Pgam2 PE = 1 SV = 3	O70250	29 kDa	69.379	34.323
Cluster of Prothrombin OS = Mus musculus GN = F2 PE = 1 SV = 1 (P19221)	P19221	70 kDa	53.653	20.594

Table S1. *Cont.*

Identified Proteins	Accession Number	Molecular Weight	Quantitative Value	
			**1 h**	**24 h**
Myomesin 2 OS = Mus musculus GN = Myom2 PE = 2 SV = 1	Q14BI5	165 kDa	14.801	72.079
Parvalbumin alpha OS = Mus musculus GN = Pvalb PE = 1 SV = 3	P32848	12 kDa	58.279	36.612
Protein Agl OS = Mus musculus GN = Agl PE = 4 SV = 1	F8VPN4	174 kDa	60.129	0
Ig mu chain C region OS = Mus musculus GN = Ighm PE = 1 SV = 2	P01872	50 kDa	49.028	32.035
Ig kappa chain C region OS = Mus musculus PE = 1 SV = 1	P01837	12 kDa	41.628	32.035
Cluster of Heat shock cognate 71 kDa protein OS = Mus musculus GN = Hspa8 PE = 1 SV = 1 (P63017)	P63017 [4]	71 kDa	50.878	17.162
Fibrinogen beta chain OS = Mus musculus GN = Fgb PE = 2 SV = 1	Q8K0E8	55 kDa	12.951	107.55
Cluster of Heat shock protein HSP 90-beta OS = Mus musculus GN = Hsp90ab1 PE = 1 SV = 3 (P11499)	P11499 [2]	83 kDa	41.628	26.315
Afamin OS = Mus musculus GN = Afm PE = 1 SV = 2	O89020	69 kDa	34.227	35.468
Inter-alpha-trypsin inhibitor heavy chain H1 OS = Mus musculus GN = Itih1 PE = 1 SV = 2	Q61702	101 kDa	49.953	48.053
Malate dehydrogenase, mitochondrial OS = Mus musculus GN = Mdh2 PE = 1 SV = 3	P08249	36 kDa	64.754	13.729
Carbonic anhydrase 2 OS = Mus musculus GN = Ca2 PE = 1 SV = 4	P00920	29 kDa	54.578	33.179
Fibrillin-2 OS = Mus musculus GN = Fbn2 PE = 1 SV = 2	Q61555	314 kDa	0.92506	0
Aconitate hydratase, mitochondrial OS = Mus musculus GN = Aco2 PE = 1 SV = 1	Q99KI0	85 kDa	48.103	10.297
Cluster of Alpha-1-acid glycoprotein 1 OS = Mus musculus GN = Orm1 PE = 1 SV = 1 (Q60590)	Q60590 [2]	24 kDa	13.876	59.494
Beta-2-glycoprotein 1 OS = Mus musculus GN = Apoh PE = 1 SV = 1	Q01339	39 kDa	33.302	22.882
Complement factor I OS = Mus musculus GN = Cfi PE = 1 SV = 3	Q61129	67 kDa	18.501	26.315
ATP-dependent 6-phosphofructokinase, muscle type OS = Mus musculus GN = Pfkm PE = 1 SV = 3	P47857	85 kDa	67.529	0
Cluster of Obscurin OS = Mus musculus GN = Obscn PE = 2 SV = 2 (A2AAJ9)	A2AAJ9 [4]	966 kDa	17.576	0
Cluster of Phosphoglucomutase-1 OS = Mus musculus GN = Pgm1 PE = 1 SV = 4 (Q9D0F9)	Q9D0F9 [2]	61 kDa	68.454	68.647
Cluster of Desmin OS = Mus musculus GN = Des PE = 1 SV = 3 (P31001)	P31001 [2]	53 kDa	28.677	27.459
Cluster of Nesprin-1 OS = Mus musculus GN = Syne1 PE = 1 SV = 2 (Q6ZWR6)	Q6ZWR6	1010 kDa	0	0
Cluster of Glycosylphosphatidylinositol specific phospholipase D1 OS = Mus musculus GN = Gpld1 PE = 2 SV = 1 (Q8VCU2)	Q8VCU2	94 kDa	41.628	20.594
Peroxiredoxin-2 OS = Mus musculus GN = Prdx2 PE = 1 SV = 3	Q61171	22 kDa	42.553	61.782
Alpha-1B-glycoprotein OS = Mus musculus GN = A1bg PE = 1 SV = 1	Q19LI2	57 kDa	10.176	50.341
Cluster of Basement membrane-specific heparan sulfate proteoglycan core protein OS = Mus musculus GN = Hspg2 PE =	B1B0C7 [2]	469 kDa	83.255	0
Plasma protease C1 inhibitor OS = Mus musculus GN = Serping1 PE = 1 SV = 3	P97290	56 kDa	31.452	34.323
Cluster of Alpha-2-antiplasmin OS = Mus musculus GN = Serpinf2 PE = 1 SV = 1 (Q61247)	Q61247	55 kDa	34.227	14.873
Cluster of Leukemia inhibitory factor receptor OS = Mus musculus GN = Lifr PE = 1 SV = 1 (P42703)	P42703 [2]	123 kDa	33.302	42.332
Apolipoprotein A-II OS = Mus musculus GN = Apoa2 PE = 1 SV = 2	P09813	11 kDa	46.253	32.035
Serum amyloid P-component OS = Mus musculus GN = Apcs PE = 1 SV = 2	P12246	26 kDa	27.752	65.215
Histidine-rich glycoprotein OS = Mus musculus GN = Hrg PE = 1 SV = 2	Q9ESB3	59 kDa	33.302	34.323
Histone H4 OS = Mus musculus GN = Hist1h4a PE = 1 SV = 2	P62806	11 kDa	55.503	46.909
Cluster of Tubulin beta-5 chain OS = Mus musculus GN = Tubb5 PE = 1 SV = 1 (P99024)	P99024 [3]	50 kDa	49.028	26.315
Retinol-binding protein 4 OS = Mus musculus GN = Rbp4 PE = 3 SV = 1	H7BWY6	28 kDa	38.852	30.891
Cluster of H-2 class I histocompatibility antigen, Q10 alpha chain OS = Mus musculus GN = H2-Q10 PE = 1 SV = 3 (P01898)	P01898 [5]	37 kDa	17.576	13.729

Table S1. *Cont.*

Identified Proteins	Accession Number	Molecular Weight	Quantitative Value	
			1 h	24 h
Cluster of 14-3-3 protein epsilon OS = Mus musculus GN = Ywhae PE = 1 SV = 1 (P62259)	P62259	29 kDa	41.628	33.179
Cluster of Plectin OS = Mus musculus GN = Plec PE = 1 SV = 3 (Q9QXS1)	Q9QXS1 [4]	534 kDa	15.726	0
Cluster of von Willebrand factor (Fragment) OS = Mus musculus GN = Vwf PE = 4 SV = 1 (S4R195)	S4R195 [2]	42 kDa	64.754	45.765
Cluster of Glycerol-3-phosphate dehydrogenase [NAD(+)], cytoplasmic OS = Mus musculus GN = Gpd1 PE = 1 SV = 3 (P137	P13707	38 kDa	48.103	34.323
Cluster of Glucose-6-phosphate isomerase OS = Mus musculus GN = Gpi PE = 1 SV = 4 (P06745)	P06745	63 kDa	30.527	17.162
Cluster of Tubulin alpha-1C chain OS = Mus musculus GN = Tuba1c PE = 1 SV = 1 (P68373)	P68373 [3]	50 kDa	31.452	17.162
Cluster of Nucleoside diphosphate kinase OS = Mus musculus GN = Gm20390 PE = 3 SV = 1 (E9PZF0)	E9PZF0 [2]	30 kDa	15.726	18.306
Isoform 3 of Sulfhydryl oxidase 1 OS = Mus musculus GN = Qsox1	Q8BND5-3	63 kDa	49.953	35.468
Inter-alpha-trypsin inhibitor heavy chain H3 OS = Mus musculus GN = Itih3 PE = 1 SV = 3	Q61704	99 kDa	10.176	46.909
Vitronectin OS = Mus musculus GN = Vtn PE = 1 SV = 2	P29788	55 kDa	18.501	10.297
Transitional endoplasmic reticulum ATPase OS = Mus musculus GN = Vcp PE = 1 SV = 4	Q01853	89 kDa	24.977	14.873
Cluster of Myosin regulatory light chain 2, skeletal muscle isoform OS = Mus musculus GN = Mylpf PE = 1 SV = 3 (P97457)	P97457	19 kDa	46.253	74.367
Cluster of Peptidyl-prolyl cis-trans isomerase A OS = Mus musculus GN = Ppia PE = 1 SV = 2 (P17742)	P17742	18 kDa	22.201	24.026
Cluster of Filamin, alpha OS = Mus musculus GN = Flna PE = 1 SV = 1 (B7FAU9)	B7FAU9	280 kDa	14.801	18.306
Cluster of Glutathione S-transferase Mu 2 OS = Mus musculus GN = Gstm2 PE = 1 SV = 2 (P15626)	P15626 [3]	26 kDa	12.951	12.585
Cluster of Epidermal growth factor receptor OS = Mus musculus GN = Egfr PE = 1 SV = 1 (Q01279)	Q01279	135 kDa	16.651	91.529
Histone H3.1 OS = Mus musculus GN = Hist1h3a PE = 1 SV = 2	P68433	15 kDa	18.501	11.441
Cluster of Clathrin heavy chain 1 OS = Mus musculus GN = Cltc PE = 1 SV = 3 (Q68FD5)	Q68FD5	192 kDa	16.651	91.529
Elongation factor 2 OS = Mus musculus GN = Eef2 PE = 1 SV = 2	P58252	95 kDa	11.101	45.765
Malate dehydrogenase, cytoplasmic OS = Mus musculus GN = Mdh1 PE = 1 SV = 3	P14152	37 kDa	42.553	80.088
Cluster of Histone H2B type 1-M OS = Mus musculus GN = Hist1h2bm PE = 1 SV = 2 (P10854)	P10854 [3]	14 kDa	46.253	29.747
Talin-1 OS = Mus musculus GN = Tln1 PE = 1 SV = 2	P26039	270 kDa	83.255	33.179
Cluster of Cytochrome c, somatic OS = Mus musculus GN = Cycs PE = 1 SV = 2 (P62897)	P62897	12 kDa	26.827	16.018
Cluster of Protein DJ-1 OS = Mus musculus GN = Park7 PE = 1 SV = 1 (Q99LX0)	Q99LX0	20 kDa	16.651	10.297
Cluster of Protein Mybpc1 OS = Mus musculus GN = Mybpc1 PE = 2 SV = 1 (Q6P6L5)	Q6P6L5	126 kDa	16.651	0
Carboxypeptidase N subunit 2 OS = Mus musculus GN = Cpn2 PE = 1 SV = 2	Q9DBB9	60 kDa	23.126	11.441
Cluster of Protein Ighg2c OS = Mus musculus GN = Ighg2c PE = 4 SV = 1 (F6TQW2)	F6TQW2	44 kDa	26.827	49.197
Cluster of Creatine kinase S-type, mitochondrial OS = Mus musculus GN = Ckmt2 PE = 1 SV = 1 (Q6P8J7)	Q6P8J7	47 kDa	16.651	11.441
Carbonic anhydrase 1 OS = Mus musculus GN = Ca1 PE = 2 SV = 4	P13634	28 kDa	37.927	21.738
Cluster of Glutathione peroxidase 3 OS = Mus musculus GN = Gpx3 PE = 2 SV = 2 (P46412)	P46412	25 kDa	29.602	27.459
Cluster of Proteoglycan 4 OS = Mus musculus GN = Prg4 PE = 4 SV = 1 (E9QQ17)	E9QQ17 [4]	111 kDa	18.501	12.585
Isoform 2 of Myomesin-1 OS = Mus musculus GN = Myom1	Q62234-2	175 kDa	64.754	11.441
Vinculin OS = Mus musculus GN = Vcl PE = 1 SV = 4	Q64727	117 kDa	16.651	19.45
Cluster of Ubiquitin-40S ribosomal protein S27a OS = Mus musculus GN = Rps27a PE = 1 SV = 2 (P62983)	P62983 [2]	18 kDa	74.005	26.315
Cluster of Cytoplasmic dynein 1 heavy chain 1 OS = Mus musculus GN = Dync1h1 PE = 1 SV = 2 (Q9JHU4)	Q9JHU4	532 kDa	12.026	11.441

Table S1. *Cont.*

Identified Proteins	Accession Number	Molecular Weight	Quantitative Value	
			**1 h**	**24 h**
Collagen alpha-1(XIV) chain OS = Mus musculus GN = Col14a1 PE = 2 SV = 1	B7ZNH7	193 kDa	12.026	28.603
Plasma kallikrein OS = Mus musculus GN = Klkb1 PE = 1 SV = 2	P26262	71 kDa	23.126	91.529
Fetuin-B OS = Mus musculus GN = Fetub PE = 1 SV = 1	Q9QXC1	43 kDa	17.576	11.441
Peroxiredoxin-1 OS = Mus musculus GN = Prdx1 PE = 1 SV = 1	P35700	22 kDa	26.827	19.45
Cluster of Clusterin OS = Mus musculus GN = Clu PE = 1 SV = 1 (Q06890)	Q06890	52 kDa	15.726	17.162
Complement component C8 gamma chain OS = Mus musculus GN = C8g PE = 1 SV = 1	Q8VCG4	23 kDa	30.527	32.035
Cluster of Four and a half LIM domains 1, isoform CRA_b OS = Mus musculus GN = Fhl1 PE = 1 SV = 1 (A2AEX8)	A2AEX8 [2]	34 kDa	32.377	0
Fatty acid-binding protein, adipocyte OS = Mus musculus GN = Fabp4 PE = 1 SV = 3	P04117	15 kDa	24.051	19.45
Thrombospondin-1 OS = Mus musculus GN = Thbs1 PE = 1 SV = 1	P35441	130 kDa	64.754	11.441
Corticosteroid-binding globulin OS = Mus musculus GN = Serpina6 PE = 1 SV = 1	Q06770	45 kDa	29.602	10.297
Maltase-glucoamylase OS = Mus musculus GN = Mgam PE = 2 SV = 1	B5THE2	209 kDa	24.051	13.729
Aspartate aminotransferase, mitochondrial OS = Mus musculus GN = Got2 PE = 1 SV = 1	P05202	47 kDa	25.902	45.765
Elongation factor 1-gamma OS = Mus musculus GN = Eef1g PE = 1 SV = 3	Q9D8N0	50 kDa	15.726	68.647
Cluster of Ig heavy chain V region AC38 205.12 OS = Mus musculus PE = 1 SV = 1 (P06330)	P06330 [3]	13 kDa	12.951	12.585
Protein S100-A9 OS = Mus musculus GN = S100a9 PE = 1 SV = 3	P31725	13 kDa	0.92506	21.738
Cluster of Ras-related protein Rab-14 OS = Mus musculus GN = Rab14 PE = 3 SV = 1 (A2AL34)	A2AL34 [7]	17 kDa	92.506	68.647
Cluster of Myosin light chain 1/3, skeletal muscle isoform OS = Mus musculus GN = Myl1 PE = 1 SV = 2 (P05977)	P05977 [2]	21 kDa	10.176	59.494
Ryanodine receptor 1 OS = Mus musculus GN = Ryr1 PE = 1 SV = 1	E9PZQ0	565 kDa	18.501	0
Lumican OS = Mus musculus GN = Lum PE = 1 SV = 2	P51885	38 kDa	11.101	17.162
Protein Krt78 OS = Mus musculus GN = Krt78 PE = 4 SV = 1	E9Q0F0	112 kDa	15.726	11.441
Troponin C, skeletal muscle OS = Mus musculus GN = Tnnc2 PE = 1 SV = 2	P20801	18 kDa	12.951	32.035
ATP-binding cassette sub-family A member 8-B OS = Mus musculus GN = Abca8b PE = 2 SV = 2	Q8K440	183 kDa	92.506	57.206
Protein AMBP OS = Mus musculus GN = Ambp PE = 2 SV = 2	Q07456	39 kDa	18.501	29.747
Cluster of Ig kappa chain V-V region HP 123E6 OS = Mus musculus PE = 1 SV = 1 (P01646)	P01646	12 kDa	15.726	19.45
Myoglobin OS = Mus musculus GN = Mb PE = 1 SV = 3	P04247	17 kDa	12.951	11.441
Cluster of Complement component C9 OS = Mus musculus GN = C9 PE = 1 SV = 2 (P06683)	P06683	62 kDa	55.503	22.882
Myosin-9 OS = Mus musculus GN = Myh9 PE = 1 SV = 4	Q8VDD5	226 kDa	64.754	30.891
Troponin I, fast skeletal muscle OS = Mus musculus GN = Tnni2 PE = 1 SV = 2	P13412	21 kDa	46.253	16.018
Complement component C8 alpha chain OS = Mus musculus GN = C8a PE = 2 SV = 1	Q8K182	66 kDa	15.726	57.206
Cluster of Citrate synthase, mitochondrial OS = Mus musculus GN = Cs PE = 1 SV = 1 (Q9CZU6)	Q9CZU6 [2]	52 kDa	55.503	11.441
Transketolase OS = Mus musculus GN = Tkt PE = 1 SV = 1	P40142	68 kDa	37.002	14.873
Myeloid bactenecin (F1) OS = Mus musculus GN = Ngp PE = 2 SV = 1	O08692	19 kDa	0	50.341
14-3-3 protein sigma OS = Mus musculus GN = Sfn PE = 1 SV = 2	O70456	28 kDa	37.002	35.468
Kelch-like protein 41 OS = Mus musculus GN = Klhl41 PE = 1 SV = 1	A2AUC9	68 kDa	64.754	0
Collagen alpha-2(I) chain OS = Mus musculus GN = Col1a2 PE = 2 SV = 2	Q01149	130 kDa	15.726	57.206

Table S1. *Cont.*

Identified Proteins	Accession Number	Molecular Weight	Quantitative Value	
			**1 h**	**24 h**
Ig kappa chain V-V region K2 (Fragment) OS = Mus musculus PE = 1 SV = 1	P01635	13 kDa	83.255	29.747
Xanthine dehydrogenase/oxidase OS = Mus musculus GN = Xdh PE = 1 SV = 5	Q00519	147 kDa	0.92506	34.323
Alpha-2-macroglobulin-P OS = Mus musculus GN = A2mp PE = 2 SV = 2	Q6GQT1	164 kDa	25.902	57.206
Cluster of Isoform Short of 14-3-3 protein beta/alpha OS = Mus musculus GN = Ywhab (Q9CQV8-2)	Q9CQV8-2 [2]	28 kDa	46.253	48.053
Cluster of Laminin subunit gamma-1 OS = Mus musculus GN = Lamc1 PE = 1 SV = 2 (P02468)	P02468 [2]	177 kDa	0.92506	45.765
Serum amyloid A-4 protein OS = Mus musculus GN = Saa4 PE = 1 SV = 2	P31532	15 kDa	12.951	11.441
Elongation factor 1-alpha 2 OS = Mus musculus GN = Eef1a2 PE = 1 SV = 1	P62631	50 kDa	42.553	22.882
Cluster of Flavin reductase (NADPH) OS = Mus musculus GN = Blvrb PE = 2 SV = 3 (Q923D2)	Q923D2	22 kDa	74.005	21.738
Zinc-alpha-2-glycoprotein OS = Mus musculus GN = Azgp1 PE = 1 SV = 2	Q64726	35 kDa	92.506	16.018
Heparin cofactor 2 OS = Mus musculus GN = Serpind1 PE = 1 SV = 1	P49182	54 kDa	27.752	22.882
Cluster of Bisphosphoglycerate mutase OS = Mus musculus GN = Bpgm PE = 2 SV = 2 (P15327)	P15327 [2]	30 kDa	92.506	13.729
Profilin-1 OS = Mus musculus GN = Pfn1 PE = 1 SV = 2	P62962	15 kDa	11.101	26.315
Cluster of Angiotensinogen OS = Mus musculus GN = Agt PE = 2 SV = 1 (Q3UTR7)	Q3UTR7	53 kDa	83.255	21.738
Cluster of Ubiquitin-like modifier-activating enzyme 1 OS = Mus musculus GN = Uba1 PE = 1 SV = 1 (Q02053)	Q02053	118 kDa	12.026	45.765
Cluster of 14-3-3 protein zeta/delta OS = Mus musculus GN = Ywhaz PE = 1 SV = 1 (P63101)	P63101	28 kDa	20.351	37.756
Cluster of Histone H2A OS = Mus musculus GN = Hist1h2aa PE = 2 SV = 1 (Q8CGP4)	Q8CGP4 [2]	14 kDa	27.752	34.323
Cluster of Serum paraoxonase/arylesterase 1 OS = Mus musculus GN = Pon1 PE = 1 SV = 2 (P52430)	P52430	40 kDa	20.351	10.297
Phosphatidylethanolamine-binding protein 1 OS = Mus musculus GN = Pebp1 PE = 1 SV = 3	P70296	21 kDa	13.876	57.206
Beta-2-microglobulin OS = Mus musculus GN = B2m PE = 1 SV = 2	P01887	14 kDa	74.005	17.162
Cluster of MCG48959 OS = Mus musculus GN = Prdx6b PE = 2 SV = 1 (Q8BG37)	Q8BG37 [2]	25 kDa	74.005	12.585
Cluster of Peroxiredoxin-5, mitochondrial OS = Mus musculus GN = Prdx5 PE = 1 SV = 2 (P99029)	P99029 [2]	22 kDa	83.255	17.162
Spectrin beta chain, non-erythrocytic 1 OS = Mus musculus GN = Sptbn1 PE = 1 SV = 2	Q62261	274 kDa	18.501	45.765
Cluster of Glutathione S-transferase P 1 OS = Mus musculus GN = Gstp1 PE = 1 SV = 2 (P19157)	P19157	24 kDa	15.726	80.088
Mannose-binding protein A OS = Mus musculus GN = Mbl1 PE = 2 SV = 1	P39039	25 kDa	28.677	11.441
Coagulation factor XII OS = Mus musculus GN = F12 PE = 2 SV = 2	Q80YC5	66 kDa	64.754	68.647
Apolipoprotein M OS = Mus musculus GN = Apom PE = 1 SV = 1	Q9Z1R3	21 kDa	10.176	34.323
Cluster of Transferrin receptor protein 1 OS = Mus musculus GN = Tfrc PE = 1 SV = 1 (Q62351)	Q62351	86 kDa	12.026	22.882
Cluster of Aspartate aminotransferase, cytoplasmic OS = Mus musculus GN = Got1 PE = 1 SV = 3 (P05201)	P05201	46 kDa	11.101	11.441
Cluster of Protein Col6a3 OS = Mus musculus GN = Col6a3 PE = 1 SV = 2 (E9PWQ3)	E9PWQ3	354 kDa	55.503	0
Collagen alpha-1(I) chain OS = Mus musculus GN = Col1a1 PE = 1 SV = 4	P11087	138 kDa	14.801	57.206
Apolipoprotein D OS = Mus musculus GN = Apod PE = 2 SV = 1	P51910	22 kDa	18.501	12.585
Cluster of Spectrin alpha chain, non-erythrocytic 1 OS = Mus musculus GN = Sptan1 PE = 1 SV = 4 (P16546)	P16546 [2]	285 kDa	18.501	34.323
LIM domain-binding protein 3 OS = Mus musculus GN = Ldb3 PE = 4 SV = 1	E9PYJ9 (+2)	72 kDa	15.726	0
Leucine-rich HEV glycoprotein OS = Mus musculus GN = Lrg1 PE = 2 SV = 1	Q91XL1	37 kDa	64.754	27.459
Cluster of Major urinary protein 8 OS = Mus musculus GN = Mup10 PE = 2 SV = 1 (A2BIN1)	A2BIN1	21 kDa	55.503	13.729
Isoform 2 of Ig gamma-3 chain C region OS = Mus musculus	P03987-2	36 kDa	74.005	80.088

Table S1. *Cont.*

Identified Proteins	Accession Number	Molecular Weight	Quantitative Value	
			1 h	24 h
Glutathione peroxidase 1 OS = Mus musculus GN = Gpx1 PE = 1 SV = 2	P11352	22 kDa	83.255	13.729
Fatty acid-binding protein, heart OS = Mus musculus GN = Fabp3 PE = 1 SV = 5	P11404	15 kDa	14.801	0
Cluster of ADP-ribosylation factor 4 OS = Mus musculus GN = Arf4 PE = 1 SV = 2 (P61750)	P61750 [2]	20 kDa	10.176	91.529
Thrombospondin-4 OS = Mus musculus GN = Thbs4 PE = 1 SV = 1	Q9Z1T2	106 kDa	64.754	45.765
Insulin-like growth factor-binding protein complex acid labile subunit OS = Mus musculus GN = Igfals PE = 2 SV = 1	P70389	67 kDa	10.176	11.441
Isoform 4 of Periostin OS = Mus musculus GN = Postn	Q62009-4	87 kDa	0.92506	68.647
Complement component C8 beta chain OS = Mus musculus GN = C8b PE = 1 SV = 1	Q8BH35	66 kDa	55.503	22.882
Cluster of Proteasome subunit alpha type-7 OS = Mus musculus GN = Psma7 PE = 1 SV = 1 (Q9Z2U0)	Q9Z2U0	28 kDa	64.754	13.729
Cluster of Elongation factor 1-alpha 1 OS = Mus musculus GN = Eef1a1 PE = 1 SV = 3 (P10126)	P10126	50 kDa	31.452	28.603
Pigment epithelium-derived factor OS = Mus musculus GN = Serpinf1 PE = 1 SV = 2	P97298	46 kDa	27.752	80.088
C-type lectin domain family 3, member b OS = Mus musculus GN = Clec3b PE = 2 SV = 1	Q8CFZ6	22 kDa	64.754	80.088
Carboxypeptidase N catalytic chain OS = Mus musculus GN = Cpn1 PE = 2 SV = 1	Q9JJN5	52 kDa	12.026	91.529
UTP--glucose-1-phosphate uridylyltransferase OS = Mus musculus GN = Ugp2 PE = 2 SV = 3	Q91ZJ5	57 kDa	74.005	11.441
Cluster of Protein disulfide-isomerase OS = Mus musculus GN = P4hb PE = 1 SV = 2 (P09103)	P09103	57 kDa	27.752	68.647
Nidogen-2 OS = Mus musculus GN = Nid2 PE = 1 SV = 2	O88322	154 kDa	18.501	0
Spectrin alpha chain, erythrocytic 1 OS = Mus musculus GN = Spta1 PE = 2 SV = 3	P08032	280 kDa	0	34.323
Collagen alpha-1(III) chain OS = Mus musculus GN = Col3a1 PE = 1 SV = 4	P08121	139 kDa	12.026	22.882
C-reactive protein OS = Mus musculus GN = Crp PE = 2 SV = 2	P14847	25 kDa	64.754	19.45
Probable C->U-editing enzyme APOBEC-2 OS = Mus musculus GN = Apobec2 PE = 1 SV = 1	Q9WV35	26 kDa	83.255	91.529
Cluster of Collagen alpha-2(IV) chain OS = Mus musculus GN = Col4a2 PE = 2 SV = 4 (P08122)	P08122	167 kDa	37.002	0
Isoform Cytoplasmic of Fumarate hydratase, mitochondrial OS = Mus musculus GN = Fh	P97807-2	50 kDa	46.253	68.647
Cluster of Selenoprotein P OS = Mus musculus GN = Sepp1 PE = 2 SV = 3 (P70274)	P70274	43 kDa	0.92506	11.441
Alpha-crystallin B chain OS = Mus musculus GN = Cryab PE = 1 SV = 2	P23927	20 kDa	17.576	0
Nucleolar pre-ribosomal-associated protein 1 OS = Mus musculus GN = Urb1 PE = 4 SV = 1	E9PU96 (+1)	255 kDa	18.501	11.441
Cluster of Adenylate kinase isoenzyme 1 OS = Mus musculus GN = Ak1 PE = 1 SV = 1 (Q9R0Y5)	Q9R0Y5 [2]	22 kDa	20.351	22.882
Protein 9530053A07Rik OS = Mus musculus GN = 9530053A07Rik PE = 4 SV = 1	E9PVG8	280 kDa	18.501	11.441
Complement C5 OS = Mus musculus GN = C5 PE = 1 SV = 2	P06684	189 kDa	27.752	0
Cluster of GTP-binding nuclear protein Ran, testis-specific isoform OS = Mus musculus GN = Rasl2-9 PE = 2 SV = 1 (Q61820	Q61820 [2]	24 kDa	11.101	80.088
Sarcalumenin OS = Mus musculus GN = Srl PE = 1 SV = 1	Q7TQ48	99 kDa	10.176	0
Rho GDP-dissociation inhibitor 1 OS = Mus musculus GN = Arhgdia PE = 1 SV = 3	Q99PT1	23 kDa	13.876	11.441
Cluster of Ig kappa chain V-V region L6 (Fragment) OS = Mus musculus PE = 4 SV = 1 (P01638)	P01638	13 kDa	46.253	10.297
Superoxide dismutase [Cu-Zn] OS = Mus musculus GN = Sod1 PE = 1 SV = 2	P08228	16 kDa	27.752	57.206
Isocitrate dehydrogenase [NADP], mitochondrial OS = Mus musculus GN = Idh2 PE = 1 SV = 3	P54071	51 kDa	18.501	0
Cluster of Ig kappa chain V-II region 26-10 OS = Mus musculus PE = 1 SV = 1 (P01631)	P01631	12 kDa	83.255	80.088
Cluster of Ig heavy chain V region MOPC 173 OS = Mus musculus PE = 1 SV = 1 (P01812)	P01812 [3]	13 kDa	74.005	11.441

Table S1. Cont.

Identified Proteins	Accession Number	Molecular Weight	Quantitative Value	
			1 h	24 h
Complement C1q subcomponent subunit A OS = Mus musculus GN = C1qa PE = 1 SV = 2	P98086	26 kDa	74.005	10.297
Oxidation resistance protein 1 OS = Mus musculus GN = C7 PE = 4 SV = 2	D3YXF5	93 kDa	37.002	11.441
Fatty acid-binding protein, epidermal OS = Mus musculus GN = Fabp5 PE = 1 SV = 3	Q05816	15 kDa	18.501	14.873
Cluster of L-lactate dehydrogenase B chain OS = Mus musculus GN = Ldhb PE = 1 SV = 2 (P16125)	P16125	37 kDa	64.754	80.088
Cluster of Ig heavy chain V region B1-8/186-2 OS = Mus musculus GN = Ighv1-72 PE = 1 SV = 1 (P01751)	P01751 [2]	15 kDa	37.002	0
Cluster of Moesin OS = Mus musculus GN = Msn PE = 1 SV = 3 (P26041)	P26041	68 kDa	0	80.088
Cluster of Isoform 2 of Myc box-dependent-interacting protein 1 OS = Mus musculus GN = Bin1 (O08539-2)	O08539-2 [2]	48 kDa	83.255	0
Mannose-binding protein C OS = Mus musculus GN = Mbl2 PE = 2 SV = 2	P41317	26 kDa	11.101	91.529
Cluster of Protein Z-dependent protease inhibitor OS = Mus musculus GN = Serpina10 PE = 1 SV = 1 (Q8R121)	Q8R121	52 kDa	0.92506	34.323
Cluster of C4b-binding protein OS = Mus musculus GN = C4bpa PE = 1 SV = 3 (P08607)	P08607	52 kDa	64.754	22.882
Nidogen-1 OS = Mus musculus GN = Nid1 PE = 1 SV = 2	P10493	137 kDa	18.501	0
Cluster of Fibulin-1 OS = Mus musculus GN = Fbln1 PE = 1 SV = 2 (Q08879)	Q08879	78 kDa	0	0
Cluster of Tropomyosin alpha-3 chain OS = Mus musculus GN = Tpm3 PE = 3 SV = 1 (E9Q5J9)	E9Q5J9 [5]	33 kDa	0.92506	22.882
Cluster of Complement C1s-A subcomponent OS = Mus musculus GN = C1sa PE = 2 SV = 2 (Q8CG14)	Q8CG14 [3]	77 kDa	74.005	22.882
Transgelin-2 OS = Mus musculus GN = Tagln2 PE = 1 SV = 4	Q9WVA4	22 kDa	55.503	16.018
Ig kappa chain V19-17 OS = Mus musculus GN = Igk-V19-17 PE = 1 SV = 1	P01633	16 kDa	18.501	27.459
Proteasome subunit alpha type-6 OS = Mus musculus GN = Psma6 PE = 1 SV = 1	Q9QUM9	27 kDa	37.002	11.441
Protein Sptbn2 OS = Mus musculus GN = Sptbn2 PE = 1 SV = 1	Q68FG2	271 kDa	0	22.882
Proteasome subunit beta type-5 OS = Mus musculus GN = Psmb5 PE = 1 SV = 3	O55234	29 kDa	14.801	34.323
Ig alpha chain C region OS = Mus musculus PE = 1 SV = 1	P01878	37 kDa	12.026	91.529
Cluster of Purine nucleoside phosphorylase OS = Mus musculus GN = Pnp PE = 1 SV = 2 (P23492)	P23492 [2]	32 kDa	37.002	91.529
Succinyl-CoA ligase [ADP-forming] subunit beta, mitochondrial OS = Mus musculus GN = Sucla2 PE = 1 SV = 2	Q9Z2I9	50 kDa	0	0
14-3-3 protein gamma OS = Mus musculus GN = Ywhag PE = 1 SV = 2	P61982	28 kDa	49.953	33.179
Myotilin OS = Mus musculus GN = Myot PE = 1 SV = 1	Q9JIF9	55 kDa	46.253	0
Complement C1q subcomponent subunit B OS = Mus musculus GN = C1qb PE = 1 SV = 2	P14106	27 kDa	10.176	80.088
Prelamin-A/C OS = Mus musculus GN = Lmna PE = 1 SV = 2	P48678	74 kDa	0.92506	57.206
Isocitrate dehydrogenase [NADP] cytoplasmic OS = Mus musculus GN = Idh1 PE = 1 SV = 2	O88844	47 kDa	18.501	22.882
ATP-citrate synthase OS = Mus musculus GN = Acly PE = 1 SV = 1	Q91V92	120 kDa	64.754	22.882
Cluster of Isoform 3 of Glyoxalase domain-containing protein 4 OS = Mus musculus GN = Glod4 (Q9CPV4-3)	Q9CPV4-3	31 kDa	74.005	57.206
Proteasome subunit alpha type-1 OS = Mus musculus GN = Psma1 PE = 1 SV = 1	Q9R1P4	30 kDa	46.253	57.206
Early endosome antigen 1 OS = Mus musculus GN = Eea1 PE = 1 SV = 2	Q8BL66	161 kDa	0	0
Myosin-10 OS = Mus musculus GN = Myh10 PE = 1 SV = 1	Q3UH59	233 kDa	0.92506	22.882
Neutrophil gelatinase-associated lipocalin OS = Mus musculus GN = Lcn2 PE = 1 SV = 1	P11672	23 kDa	0	11.441
Proteasome subunit beta type-4 OS = Mus musculus GN = Psmb4 PE = 1 SV = 1	P99026	29 kDa	55.503	80.088
Rho GDP-dissociation inhibitor 2 OS = Mus musculus GN = Arhgdib PE = 1 SV = 3	Q61599	23 kDa	0	11.441

Table S1. *Cont.*

Identified Proteins	Accession Number	Molecular Weight	Quantitative Value	
			1 h	24 h
Galectin OS = Mus musculus GN = Lgals7 PE = 2 SV = 1	Q9CRB1	15 kDa	18.501	68.647
Cluster of Protein C6 OS = Mus musculus GN = C6 PE = 4 SV = 1 (E9Q6D8)	E9Q6D8 [2]	104 kDa	0	11.441
40S ribosomal protein S16 OS = Mus musculus GN = Rps16 PE = 2 SV = 4	P14131	16 kDa	18.501	34.323
3-ketoacyl-CoA thiolase, mitochondrial OS = Mus musculus GN = Acaa2 PE = 1 SV = 3	Q8BWT1	42 kDa	27.752	11.441
Proteasome subunit alpha type-4 OS = Mus musculus GN = Psma4 PE = 1 SV = 1	Q9R1P0	29 kDa	55.503	91.529
Cluster of Aldose reductase OS = Mus musculus GN = Akr1b1 PE = 1 SV = 3 (P45376)	P45376	36 kDa	55.503	22.882
Protein NDRG2 OS = Mus musculus GN = Ndrg2 PE = 1 SV = 1	Q9QYG0 (+1)	41 kDa	12.951	0
Alcohol dehydrogenase class-3 OS = Mus musculus GN = Adh5 PE = 1 SV = 3	P28474	40 kDa	0.92506	11.441
Cluster of Proteasome subunit alpha type-5 OS = Mus musculus GN = Psma5 PE = 1 SV = 1 (Q9Z2U1)	Q9Z2U1	26 kDa	46.253	91.529
Cholinesterase OS = Mus musculus GN = Bche PE = 2 SV = 2	Q03311	68 kDa	0.92506	34.323
Cofilin-2 OS = Mus musculus GN = Cfl2 PE = 1 SV = 1	P45591	19 kDa	16.651	45.765
Succinate dehydrogenase [ubiquinone] iron-sulfur subunit, mitochondrial OS = Mus musculus GN = Sdhb PE = 1 SV = 1	Q9CQA3	32 kDa	0.92506	91.529
Cluster of Importin-5 OS = Mus musculus GN = Ipo5 PE = 1 SV = 3 (Q8BKC5)	Q8BKC5	124 kDa	0.92506	0
Cluster of Keratin, type I cuticular Ha5 OS = Mus musculus GN = Krt35 PE = 2 SV = 1 (Q497I4)	Q497I4	51 kDa	64.754	45.765
Cluster of Cullin-associated NEDD8-dissociated protein 1 OS = Mus musculus GN = Cand1 PE = 2 SV = 2 (Q6ZQ38)	Q6ZQ38	136 kDa	0	22.882
Enoyl-CoA delta isomerase 1, mitochondrial OS = Mus musculus GN = Eci1 PE = 1 SV = 2	P42125	32 kDa	55.503	45.765
Cluster of Eukaryotic translation initiation factor 5A-1 OS = Mus musculus GN = Eif5a PE = 1 SV = 2 (P63242)	P63242 [2]	17 kDa	46.253	45.765
Cluster of Isoform 3 of 2-oxoglutarate dehydrogenase, mitochondrial OS = Mus musculus GN = Ogdh (Q60597-3)	Q60597-3	118 kDa	0.92506	0
Cluster of Isoform 2 of Cytosol aminopeptidase OS = Mus musculus GN = Lap3 (Q9CPY7-2)	Q9CPY7-2	53 kDa	0	22.882
Peptidyl-prolyl cis-trans isomerase C OS = Mus musculus GN = Ppic PE = 1 SV = 1	P30412	23 kDa	74.005	91.529
Complement C1q subcomponent subunit C OS = Mus musculus GN = C1qc PE = 2 SV = 2	Q02105	26 kDa	92.506	45.765
Lactoylglutathione lyase OS = Mus musculus GN = Glo1 PE = 1 SV = 3	Q9CPU0	21 kDa	46.253	57.206
Cluster of Collagen alpha-1(XVIII) chain OS = Mus musculus GN = Col18a1 PE = 4 SV = 1 (E9QPX1)	E9QPX1 [2]	182 kDa	74.005	0
Adenosylhomocysteinase OS = Mus musculus GN = Ahcy PE = 1 SV = 3	P50247	48 kDa	0	57.206
Cluster of Adiponectin OS = Mus musculus GN = Adipoq PE = 1 SV = 2 (Q60994)	Q60994	27 kDa	92.506	68.647
Electron transfer flavoprotein subunit alpha, mitochondrial OS = Mus musculus GN = Etfa PE = 1 SV = 2	Q99LC5	35 kDa	13.876	0
Coagulation factor X OS = Mus musculus GN = F10 PE = 1 SV = 1	O88947	54 kDa	27.752	11.441
60S ribosomal protein L12 OS = Mus musculus GN = Rpl12 PE = 1 SV = 2	P35979	18 kDa	27.752	45.765
Electron transfer flavoprotein subunit beta OS = Mus musculus GN = Etfb PE = 1 SV = 3	Q9DCW4	28 kDa	27.752	0
Ras GTPase-activating-like protein IQGAP1 OS = Mus musculus GN = Iqgap1 PE = 1 SV = 2	Q9JKF1	189 kDa	0	45.765
Cluster of Proteasome subunit beta type-3 OS = Mus musculus GN = Psmb3 PE = 1 SV = 1 (Q9R1P1)	Q9R1P1	23 kDa	64.754	91.529
Cluster of Apolipoprotein C-III OS = Mus musculus GN = Apoc3 PE = 1 SV = 2 (P33622)	P33622	11 kDa	92.506	11.441
Isoform 2 of Acetyl-CoA carboxylase 1 OS = Mus musculus GN = Acaca	Q5SWU9-2	270 kDa	18.501	0
Proteasome subunit alpha type-3 OS = Mus musculus GN = Psma3 PE = 1 SV = 3	O70435	28 kDa	55.503	57.206
Transgelin OS = Mus musculus GN = Tagln PE = 1 SV = 3	P37804	23 kDa	37.002	34.323

Table S1. *Cont.*

Identified Proteins	Accession Number	Molecular Weight	Quantitative Value	
			**1 h**	**24 h**
Adenylyl cyclase-associated protein 1 OS = Mus musculus GN = Cap1 PE = 1 SV = 4	P40124	52 kDa	64.754	34.323
CD5 antigen-like OS = Mus musculus GN = Cd5l PE = 1 SV = 3	Q9QWK4	39 kDa	0.92506	34.323
Chloride intracellular channel protein 1 OS = Mus musculus GN = Clic1 PE = 1 SV = 3	Q9Z1Q5	27 kDa	37.002	10.297
Disintegrin and metalloproteinase domain-containing protein 21 OS = Mus musculus GN = Adam21 PE = 2 SV = 1	Q9JI76	81 kDa	37.002	22.882
Adenylosuccinate synthetase isozyme 1 OS = Mus musculus GN = Adssl1 PE = 3 SV = 1	J3QN31 (+2)	53 kDa	92.506	0
Cluster of Isoform 3 of F-actin-capping protein subunit beta OS = Mus musculus GN = Capzb (P47757-4)	P47757-4	34 kDa	55.503	80.088
Guanine nucleotide-binding protein subunit beta-2-like 1 OS = Mus musculus GN = Gnb2l1 PE = 1 SV = 3	P68040	35 kDa	37.002	11.441
Transaldolase OS = Mus musculus GN = Taldo1 PE = 1 SV = 2	Q93092	37 kDa	0	0
Cluster of Ferritin OS = Mus musculus GN = Ftl1 PE = 2 SV = 1 (Q9CPX4)	Q9CPX4	21 kDa	37.002	18.306
Guanine deaminase OS = Mus musculus GN = Gda PE = 1 SV = 1	Q9R111	51 kDa	0.92506	45.765
Cluster of Protein Gm20431 OS = Mus musculus GN = Gm20431 PE = 4 SV = 1 (E9PY39)	E9PY39	42 kDa	18.501	34.323
Macrophage colony-stimulating factor 1 receptor OS = Mus musculus GN = Csf1r PE = 1 SV = 3	P09581	109 kDa	83.255	11.441
Cluster of Dihydropteridine reductase OS = Mus musculus GN = Qdpr PE = 1 SV = 2 (Q8BVI4)	Q8BVI4	26 kDa	55.503	22.882
Transient receptor potential cation channel subfamily M member 2 OS = Mus musculus GN = Trpm2 PE = 2 SV = 1	Q5KTC0	172 kDa	0	22.882
Cluster of Lysozyme C-2 OS = Mus musculus GN = Lyz2 PE = 1 SV = 2 (P08905)	P08905	17 kDa	27.752	57.206
Cluster of EGF-containing fibulin-like extracellular matrix protein 1 OS = Mus musculus GN = Efemp1 PE = 2 SV = 1 (Q8BPB)	Q8BPB5	55 kDa	0	0
Cluster of Collagen alpha-1(XV) chain OS = Mus musculus GN = Col15a1 PE = 4 SV = 1 (A2AJY2)	A2AJY2 [2]	138 kDa	55.503	0
78 kDa glucose-regulated protein OS = Mus musculus GN = Hspa5 PE = 1 SV = 3	P20029	72 kDa	83.255	57.206
14-3-3 protein eta OS = Mus musculus GN = Ywhah PE = 1 SV = 2	P68510	28 kDa	41.628	33.179
Cluster of Interleukin-1 receptor accessory protein OS = Mus musculus GN = Il1rap PE = 2 SV = 1 (Q3UVZ1)	Q3UVZ1	79 kDa	37.002	22.882
Cluster of Complement C1r-A subcomponent OS = Mus musculus GN = C1ra PE = 1 SV = 1 (Q8CG16)	Q8CG16	80 kDa	46.253	0
Angiotensin-converting enzyme OS = Mus musculus GN = Ace PE = 1 SV = 3	P09470	151 kDa	27.752	0
Ig kappa chain V-V region MOPC 149 OS = Mus musculus PE = 1 SV = 1	P01636	12 kDa	83.255	10.297
Cluster of Isoform 2 of Seprase OS = Mus musculus GN = Fap (P97321-2)	P97321-2 [2]	87 kDa	64.754	11.441
Cluster of HMW kininogen-II OS = Mus musculus GN = Kng2 PE = 2 SV = 1 (Q6S9I3)	Q6S9I3 [2]	71 kDa	74.005	80.088
Cluster of Adenylosuccinate lyase OS = Mus musculus GN = Adsl PE = 4 SV = 1 (E9Q242)	E9Q242	53 kDa	37.002	11.441
Cluster of Procollagen C-endopeptidase enhancer 1 OS = Mus musculus GN = Pcolce PE = 1 SV = 2 (Q61398)	Q61398	50 kDa	11.101	11.441
Thioredoxin OS = Mus musculus GN = Txn PE = 1 SV = 3	P10639	12 kDa	46.253	91.529
Coactosin-like protein OS = Mus musculus GN = Cotl1 PE = 1 SV = 3	Q9CQI6	16 kDa	0	22.882
6-phosphogluconate dehydrogenase, decarboxylating OS = Mus musculus GN = Pgd PE = 1 SV = 3	Q9DCD0	53 kDa	27.752	80.088
60 kDa heat shock protein, mitochondrial OS = Mus musculus GN = Hspd1 PE = 1 SV = 1	P63038	61 kDa	18.501	0
Cluster of Calmodulin OS = Mus musculus GN = Calm1 PE = 1 SV = 1 (Q3UKW2)	Q3UKW2	22 kDa	55.503	45.765
Cluster of Talin-2 OS = Mus musculus GN = Tln2 PE = 1 SV = 1 (E9PUM4)	E9PUM4	272 kDa	18.501	22.882
ATP synthase subunit beta, mitochondrial OS = Mus musculus GN = Atp5b PE = 1 SV = 2	P56480	56 kDa	0.92506	80.088
Cluster of Ig kappa chain V-III region PC 2880/PC 1229 OS = Mus musculus PE = 1 SV = 1 (P01654)	P01654 [2]	12 kDa	74.005	16.018

Table S1. *Cont.*

Identified Proteins	Accession Number	Molecular Weight	Quantitative Value	
			1 h	24 h
Cluster of Chloride intracellular channel protein 4 OS = Mus musculus GN = Clic4 PE = 1 SV = 3 (Q9QYB1)	Q9QYB1	29 kDa	0.92506	80.088
Protein NipSnap homolog 2 OS = Mus musculus GN = Gbas PE = 2 SV = 1	O55126	33 kDa	92.506	0
Peptidyl-prolyl cis-trans isomerase B OS = Mus musculus GN = Ppib PE = 1 SV = 2	P24369	24 kDa	27.752	45.765
Proteasome subunit alpha type-2 OS = Mus musculus GN = Psma2 PE = 1 SV = 3	P49722	26 kDa	46.253	68.647
Phosphoglycerate mutase 1 OS = Mus musculus GN = Pgam1 PE = 1 SV = 3	Q9DBJ1	29 kDa	16.651	13.729
Filamin-B OS = Mus musculus GN = Flnb PE = 1 SV = 3	Q80X90	278 kDa	10.176	22.882
Cluster of Rab GDP dissociation inhibitor beta OS = Mus musculus GN = Gdi2 PE = 1 SV = 1 (Q61598)	Q61598	51 kDa	18.501	0
Cluster of Collagen alpha-1(XII) chain OS = Mus musculus GN = Col12a1 PE = 4 SV = 1 (E9PX70)	E9PX70 [3]	334 kDa	0	0
Cluster of Chitinase-like protein 3 OS = Mus musculus GN = Chil3 PE = 1 SV = 2 (O35744)	O35744	44 kDa	0	12.585
Extracellular superoxide dismutase [Cu-Zn] OS = Mus musculus GN = Sod3 PE = 1 SV = 1	O09164	27 kDa	37.002	45.765
Isoform Kidney of Band 3 anion transport protein OS = Mus musculus GN = Slc4a1	P04919-2	94 kDa	27.752	11.441
Tripartite motif-containing protein 72 OS = Mus musculus GN = Trim72 PE = 1 SV = 1	Q1XH17	53 kDa	27.752	0
Plastin-2 OS = Mus musculus GN = Lcp1 PE = 1 SV = 4	Q61233	70 kDa	0	91.529
Inositol (Myo)-1(Or 4)-monophosphatase 1 OS = Mus musculus GN = Impa1 PE = 2 SV = 1	Q924B0	30 kDa	0.92506	45.765
Ig heavy chain V region 3-6 OS = Mus musculus GN = Ighv3-6 PE = 1 SV = 1	P18531	13 kDa	18.501	22.882
Pannexin-2 OS = Mus musculus GN = Panx2 PE = 2 SV = 2	Q6IMP4	75 kDa	37.002	11.441
Cluster of Tropomyosin beta chain OS = Mus musculus GN = Tpm2 PE = 1 SV = 1 (P58774)	P58774	33 kDa	0.92506	33.179
Cluster of Alpha-amylase 1 OS = Mus musculus GN = Amy1 PE = 1 SV = 2 (P00687)	P00687	58 kDa	0	22.882
Cluster of Selenium-binding protein 1 OS = Mus musculus GN = Selenbp1 PE = 1 SV = 2 (P17563)	P17563	53 kDa	0.92506	11.441
Cluster of Puromycin-sensitive aminopeptidase OS = Mus musculus GN = Npepps PE = 1 SV = 2 (Q11011)	Q11011	103 kDa	0	0
Isocitrate dehydrogenase [NAD] subunit alpha, mitochondrial OS = Mus musculus GN = Idh3a PE = 1 SV = 1	Q9D6R2	40 kDa	37.002	0
Cluster of S-formylglutathione hydrolase OS = Mus musculus GN = Esd PE = 1 SV = 1 (H3BKH6)	H3BKH6 [2]	33 kDa	64.754	34.323
Cluster of 40S ribosomal protein S3 OS = Mus musculus GN = Rps3 PE = 1 SV = 1 (P62908)	P62908	27 kDa	64.754	11.441
Cluster of Glutathione S-transferase omega-1 OS = Mus musculus GN = Gsto1 PE = 2 SV = 2 (O09131)	O09131	27 kDa	18.501	0
Cluster of Protein disulfide-isomerase A3 OS = Mus musculus GN = Pdia3 PE = 1 SV = 2 (P27773)	P27773	57 kDa	0	34.323
Cluster of 40S ribosomal protein S19 OS = Mus musculus GN = Rps19 PE = 1 SV = 3 (Q9CZX8)	Q9CZX8	16 kDa	0	34.323
Delta-aminolevulinic acid dehydratase OS = Mus musculus GN = Alad PE = 1 SV = 1	P10518	36 kDa	37.002	91.529
Galectin-1 OS = Mus musculus GN = Lgals1 PE = 1 SV = 3	P16045	15 kDa	46.253	34.323
Thioredoxin-dependent peroxide reductase, mitochondrial OS = Mus musculus GN = Prdx3 PE = 1 SV = 1	P20108	28 kDa	37.002	34.323
Serpin B5 OS = Mus musculus GN = Serpinb5 PE = 2 SV = 1	P70124	42 kDa	0	34.323
6-phosphogluconolactonase OS = Mus musculus GN = Pgls PE = 2 SV = 1	Q9CQ60	27 kDa	18.501	57.206
ES1 protein homolog, mitochondrial OS = Mus musculus GN = D10Jhu81e PE = 1 SV = 1	Q9D172	28 kDa	0.92506	11.441
Cluster of 60S acidic ribosomal protein P0 OS = Mus musculus GN = Rplp0 PE = 1 SV = 3 (P14869)	P14869	34 kDa	0.92506	0
Proteasome subunit beta type-1 OS = Mus musculus GN = Psmb1 PE = 1 SV = 1	O09061	26 kDa	0.92506	45.765
Ig kappa chain V-V region L7 (Fragment) OS = Mus musculus GN = Gm10881 PE = 1 SV = 1	P01642	13 kDa	46.253	57.206

Table S1. *Cont.*

Identified Proteins	Accession Number	Molecular Weight	Quantitative Value	
			1 h	24 h
Platelet-activating factor acetylhydrolase OS = Mus musculus GN = Pla2g7 PE = 2 SV = 2	Q60963	49 kDa	0.92506	11.441
Hepatocyte growth factor activator OS = Mus musculus GN = Hgfac PE = 1 SV = 1	Q9R098	71 kDa	64.754	0
Ribonuclease inhibitor OS = Mus musculus GN = Rnh1 PE = 1 SV = 1	Q91VI7	50 kDa	0	0
Cluster of Isoform 3 of Elongation factor 1-delta OS = Mus musculus GN = Eef1d (P57776-3)	P57776-3	73 kDa	46.253	22.882
Coagulation factor V OS = Mus musculus GN = F5 PE = 1 SV = 1	O88783	247 kDa	27.752	22.882
Isoform 2 of Complement factor D OS = Mus musculus GN = Cfd	P03953-2	28 kDa	27.752	45.765
Lactotransferrin OS = Mus musculus GN = Ltf PE = 2 SV = 4	P08071	78 kDa	0	22.882
Myosin-binding protein H OS = Mus musculus GN = Mybph PE = 1 SV = 2	P70402	53 kDa	74.005	0
Succinyl-CoA:3-ketoacid coenzyme A transferase 1, mitochondrial OS = Mus musculus GN = Oxct1 PE = 1 SV = 1	Q9D0K2	56 kDa	18.501	0
26S proteasome non-ATPase regulatory subunit 1 OS = Mus musculus GN = Psmd1 PE = 1 SV = 1	Q3TXS7	106 kDa	0.92506	0
Cluster of AP-1 complex subunit beta-1 OS = Mus musculus GN = Ap1b1 PE = 1 SV = 1 (Q5SVG4)	Q5SVG4 [3]	102 kDa	27.752	11.441
Superoxide dismutase [Mn], mitochondrial OS = Mus musculus GN = Sod2 PE = 1 SV = 3	P09671	25 kDa	0.92506	34.323
40S ribosomal protein SA OS = Mus musculus GN = Rpsa PE = 1 SV = 4	P14206	33 kDa	64.754	0
Serpin H1 OS = Mus musculus GN = Serpinh1 PE = 1 SV = 3	P19324	47 kDa	0.92506	0
Actin-related protein 2/3 complex subunit 4 OS = Mus musculus GN = Arpc4 PE = 1 SV = 3	P59999	20 kDa	27.752	80.088
Carboxymethylenebutenolidase homolog OS = Mus musculus GN = Cmbl PE = 2 SV = 1	Q8R1G2	28 kDa	27.752	0
Adenylate kinase 2, mitochondrial OS = Mus musculus GN = Ak2 PE = 1 SV = 5	Q9WTP6	26 kDa	37.002	34.323
Decorin OS = Mus musculus GN = Dcn PE = 2 SV = 1	P28654	40 kDa	0.92506	22.882
Cluster of Poly(rC)-binding protein 1 OS = Mus musculus GN = Pcbp1 PE = 1 SV = 1 (P60335)	P60335 [4]	37 kDa	64.754	0
Proteasome subunit beta type-7 OS = Mus musculus GN = Psmb7 PE = 1 SV = 1	P70195	30 kDa	0.92506	34.323
Ubiquitin-like protein ISG15 OS = Mus musculus GN = Isg15 PE = 1 SV = 4	Q64339	18 kDa	27.752	34.323
Omega-amidase NIT2 OS = Mus musculus GN = Nit2 PE = 1 SV = 1	Q9JHW2	31 kDa	37.002	22.882
Cluster of MCG1288 OS = Mus musculus GN = Gm15013 PE = 3 SV = 1 (V9GWY0)	V9GWY0	30 kDa	0.92506	0
Cluster of Insulin-degrading enzyme OS = Mus musculus GN = Ide PE = 1 SV = 1 (Q9JHR7)	Q9JHR7	118 kDa	0	11.441
Cluster of Sepiapterin reductase OS = Mus musculus GN = Spr PE = 1 SV = 1 (Q64105)	Q64105	28 kDa	55.503	11.441
Cluster of Isoform 2 of Reticulon-2 OS = Mus musculus GN = Rtn2 (O70622-2)	O70622-2	22 kDa	64.754	0
Phosphatidylcholine-sterol acyltransferase OS = Mus musculus GN = Lcat PE = 1 SV = 2	P16301	50 kDa	55.503	22.882
Tropomyosin alpha-4 chain OS = Mus musculus GN = Tpm4 PE = 2 SV = 3	Q6IRU2	28 kDa	0.92506	45.765
Cystatin E/M OS = Mus musculus GN = Cst6 PE = 2 SV = 1	Q9D1B1	17 kDa	46.253	22.882
UMP-CMP kinase OS = Mus musculus GN = Cmpk1 PE = 1 SV = 1	Q9DBP5	22 kDa	0	22.882
Proteasome subunit beta type-2 OS = Mus musculus GN = Psmb2 PE = 1 SV = 1	Q9R1P3	23 kDa	0.92506	22.882
Regenerating islet-derived protein 3-beta OS = Mus musculus GN = Reg3b PE = 1 SV = 1	P35230	19 kDa	0	57.206
Collagen alpha-1(IV) chain OS = Mus musculus GN = Col4a1 PE = 2 SV = 4	P02463	161 kDa	18.501	0
Cluster of Leukocyte elastase inhibitor A OS = Mus musculus GN = Serpinb1a PE = 1 SV = 1 (Q9D154)	Q9D154	43 kDa	0.92506	34.323
Cluster of Ig heavy chain V region H8 OS = Mus musculus PE = 1 SV = 1 (P01788)	P01788 [2]	14 kDa	37.002	11.441

Table S1. *Cont.*

Identified Proteins	Accession Number	Molecular Weight	Quantitative Value	
			**1 h**	**24 h**
Cluster of Phosphorylase b kinase regulatory subunit beta OS = Mus musculus GN = Phkb PE = 1 SV = 1 (Q7TSH2)	Q7TSH2	124 kDa	27.752	0
Dihydrolipoyl dehydrogenase, mitochondrial OS = Mus musculus GN = Dld PE = 1 SV = 2	O08749	54 kDa	0	11.441
Eukaryotic initiation factor 4A-I OS = Mus musculus GN = Eif4a1 PE = 1 SV = 1	P60843	46 kDa	18.501	34.323
Ubiquitin-conjugating enzyme E2 N OS = Mus musculus GN = Ube2n PE = 1 SV = 1	P61089	17 kDa	46.253	80.088
C-1-tetrahydrofolate synthase, cytoplasmic OS = Mus musculus GN = Mthfd1 PE = 1 SV = 4	Q922D8	101 kDa	0.92506	11.441
Low molecular weight phosphotyrosine protein phosphatase OS = Mus musculus GN = Acp1 PE = 1 SV = 3	Q9D358	18 kDa	74.005	45.765
Chondroitin sulfate proteoglycan 4 OS = Mus musculus GN = Cspg4 PE = 1 SV = 3	Q8VHY0	252 kDa	0	11.441
Isoform 2 of Myosin-11 OS = Mus musculus GN = Myh11	O08638-2	223 kDa	0.92506	34.323
Cathepsin B OS = Mus musculus GN = Ctsb PE = 1 SV = 2	P10605	37 kDa	27.752	68.647
Cytoplasmic aconitate hydratase OS = Mus musculus GN = Aco1 PE = 1 SV = 3	P28271	98 kDa	18.501	0
Transcription elongation factor B polypeptide 2 OS = Mus musculus GN = Tceb2 PE = 1 SV = 1	P62869	13 kDa	37.002	11.441
Translationally-controlled tumor protein OS = Mus musculus GN = Tpt1 PE = 1 SV = 1	P63028	19 kDa	55.503	45.765
3-hydroxyisobutyrate dehydrogenase, mitochondrial OS = Mus musculus GN = Hibadh PE = 1 SV = 1	Q99L13	35 kDa	27.752	22.882
Immunoglobulin J chain OS = Mus musculus GN = Igj PE = 2 SV = 4	P01592	18 kDa	18.501	34.323
Coagulation factor XIII A chain OS = Mus musculus GN = F13a1 PE = 2 SV = 3	Q8BH61	83 kDa	27.752	11.441
Cluster of Ras-related protein Rab-5C OS = Mus musculus GN = Rab5c PE = 1 SV = 2 (P35278)	P35278 [3]	23 kDa	46.253	34.323
Cluster of Serine/threonine-protein phosphatase PP1-gamma catalytic subunit OS = Mus musculus GN = Ppp1cc PE = 1 S	P63087	37 kDa	18.501	0
Cluster of Phospholipid hydroperoxide glutathione peroxidase, mitochondrial OS = Mus musculus GN = Gpx4 PE = 1 SV = 4	O70325	22 kDa	0.92506	0
Uncharacterized protein OS = Mus musculus GN = Gm10260 PE = 3 SV = 2	F6YVP7	18 kDa	27.752	11.441
Ubiquinone biosynthesis protein COQ9, mitochondrial OS = Mus musculus GN = Coq9 PE = 1 SV = 1	Q8K1Z0	35 kDa	37.002	0
Isochorismatase domain-containing protein 1 OS = Mus musculus GN = Isoc1 PE = 1 SV = 1	Q91V64	32 kDa	18.501	34.323
Prolyl endopeptidase OS = Mus musculus GN = Prep PE = 2 SV = 1	Q9QUR6	81 kDa	0	0
Proteasome subunit beta type-6 OS = Mus musculus GN = Psmb6 PE = 1 SV = 3	Q60692	25 kDa	27.752	22.882
Ubiquitin-conjugating enzyme E2 L3 OS = Mus musculus GN = Ube2l3 PE = 2 SV = 1	P68037	18 kDa	27.752	22.882
Cluster of Myosin regulatory light chain 12B OS = Mus musculus GN = Myl12b PE = 1 SV = 2 (Q3THE2)	Q3THE2	20 kDa	18.501	57.206
Cluster of Very long-chain specific acyl-CoA dehydrogenase, mitochondrial OS = Mus musculus GN = Acadvl PE = 1 SV = 3 (	P50544	71 kDa	0.92506	0
Endoplasmin OS = Mus musculus GN = Hsp90b1 PE = 1 SV = 2	P08113	92 kDa	0.92506	45.765
40S ribosomal protein S15a OS = Mus musculus GN = Rps15a PE = 1 SV = 2	P62245	15 kDa	18.501	11.441
Enoyl-CoA hydratase, mitochondrial OS = Mus musculus GN = Echs1 PE = 1 SV = 1	Q8BH95	31 kDa	27.752	11.441
Spectrin beta 1 OS = Mus musculus GN = Sptb PE = 1 SV = 1	Q3UGX2	268 kDa	0.92506	11.441
Cluster of Phosphorylase b kinase gamma catalytic chain, skeletal muscle/heart isoform OS = Mus musculus GN = Phkg1	P07934	45 kDa	18.501	0
Cluster of Ribosomal protein OS = Mus musculus GN = Rpl10a PE = 1 SV = 1 (Q5XJF6)	Q5XJF6	25 kDa	0.92506	0
Cluster of Actin-related protein 2/3 complex subunit 3 OS = Mus musculus GN = Arpc3 PE = 1 SV = 1 (H7BWZ3)	H7BWZ3	20 kDa	0	34.323
Cluster of Dehydrogenase/reductase SDR family member 11 OS = Mus musculus GN = Dhrs11 PE = 2 SV = 1 (Q3U0B3)	Q3U0B3	28 kDa	0	22.882
Galectin-3 OS = Mus musculus GN = Lgals3 PE = 1 SV = 3	P16110 (+1)	28 kDa	0.92506	22.882

Table S1. *Cont.*

Identified Proteins	Accession Number	Molecular Weight	Quantitative Value	
			1 h	24 h
Cluster of Transforming protein RhoA OS = Mus musculus GN = Rhoa PE = 1 SV = 1 (Q9QUI0)	Q9QUI0	22 kDa	37.002	22.882
Ig heavy chain V region 914 OS = Mus musculus PE = 1 SV = 1	P18527	11 kDa	37.002	68.647
Cluster of Isoform 3 of Reticulon-4 OS = Mus musculus GN = Rtn4 (Q99P72-1)	Q99P72-1	22 kDa	27.752	0
Cluster of Four and a half LIM domains protein 3 OS = Mus musculus GN = Fhl3 PE = 1 SV = 2 (Q9R059)	Q9R059	32 kDa	18.501	0
Heat shock protein beta-1 OS = Mus musculus GN = Hspb1 PE = 1 SV = 3	P14602 (+1)	23 kDa	37.002	45.765
Annexin A5 OS = Mus musculus GN = Anxa5 PE = 1 SV = 1	P48036	36 kDa	55.503	11.441
Importin subunit beta-1 OS = Mus musculus GN = Kpnb1 PE = 1 SV = 2	P70168	97 kDa	0	0
Sulfurtransferase OS = Mus musculus GN = Mpst PE = 1 SV = 1	Q3UW66 (+1)	33 kDa	18.501	0
Hydroxyacyl-coenzyme A dehydrogenase, mitochondrial OS = Mus musculus GN = Hadh PE = 1 SV = 2	Q61425	34 kDa	0.92506	0
Inorganic pyrophosphatase OS = Mus musculus GN = Ppa1 PE = 1 SV = 1	Q9D819	33 kDa	0.92506	11.441
Calreticulin OS = Mus musculus GN = Calr PE = 1 SV = 1	P14211	48 kDa	0	22.882
AMP deaminase 1 OS = Mus musculus GN = Ampd1 PE = 2 SV = 2	Q3V1D3	86 kDa	18.501	0
Transcobalamin-2 OS = Mus musculus GN = Tcn2 PE = 2 SV = 1	O88968	48 kDa	0.92506	68.647
Protein S100-A8 OS = Mus musculus GN = S100a8 PE = 1 SV = 3	P27005	10 kDa	0	80.088
Thimet oligopeptidase OS = Mus musculus GN = Thop1 PE = 1 SV = 1	Q8C1A5	78 kDa	0	0
Cytosolic non-specific dipeptidase OS = Mus musculus GN = Cndp2 PE = 1 SV = 1	Q9D1A2	53 kDa	0	22.882
Glycogen [starch] synthase, muscle OS = Mus musculus GN = Gys1 PE = 1 SV = 2	Q9Z1E4	84 kDa	0.92506	0
Ras suppressor protein 1 OS = Mus musculus GN = Rsu1 PE = 4 SV = 1	A2AUR7 (+1)	30 kDa	0.92506	45.765
Cluster of Proteasome activator complex subunit 1 (Fragment) OS = Mus musculus GN = Psme1 PE = 4 SV = 1 (G3UXZ5)	G3UXZ5	27 kDa	0.92506	45.765
Cluster of Annexin A2 OS = Mus musculus GN = Anxa2 PE = 1 SV = 2 (P07356)	P07356	39 kDa	0.92506	45.765
Ubiquinone biosynthesis monooxygenase COQ6 OS = Mus musculus GN = Coq6 PE = 1 SV = 1	D3YW66	47 kDa	27.752	45.765
Cluster of Protein-L-isoaspartate(D-aspartate) O-methyltransferase OS = Mus musculus GN = Pcmt1 PE = 4 SV = 1 (E0CYV0	E0CYV0 [3]	30 kDa	0.92506	0
Cluster of Catalase OS = Mus musculus GN = Cat PE = 1 SV = 4 (P24270)	P24270	60 kDa	0.92506	34.323
Cluster of PDZ and LIM domain protein 5 OS = Mus musculus GN = Pdlim5 PE = 1 SV = 4 (Q8CI51)	Q8CI51	63 kDa	27.752	0
Long-chain specific acyl-CoA dehydrogenase, mitochondrial OS = Mus musculus GN = Acadl PE = 1 SV = 2	P51174	48 kDa	0.92506	0
Inosine triphosphate pyrophosphatase OS = Mus musculus GN = Itpa PE = 1 SV = 2	Q9D892	22 kDa	18.501	68.647
Ras-related C3 botulinum toxin substrate 1 OS = Mus musculus GN = Rac1 PE = 1 SV = 1	P63001	21 kDa	46.253	68.647
60S ribosomal protein L7 OS = Mus musculus GN = Rpl7 PE = 1 SV = 2	P14148	31 kDa	18.501	11.441
Hepatocyte growth factor-like protein OS = Mus musculus GN = Mst1 PE = 3 SV = 1	E0CXN0	80 kDa	18.501	0
Cluster of Glutamate--cysteine ligase regulatory subunit OS = Mus musculus GN = Gclm PE = 2 SV = 1 (O09172)	O09172	31 kDa	18.501	22.882
Proteasome subunit beta type-8 OS = Mus musculus GN = Psmb8 PE = 1 SV = 2	P28063	30 kDa	0.92506	22.882
Cluster of 26S proteasome non-ATPase regulatory subunit 11 OS = Mus musculus GN = Psmd11 PE = 1 SV = 3 (Q8BG32)	Q8BG32	47 kDa	18.501	0
Keratin, type II cytoskeletal 72 OS = Mus musculus GN = Krt72 PE = 3 SV = 1	Q6IME9	57 kDa	37.002	0
Cluster of Ras-related protein Rap-1A OS = Mus musculus GN = Rap1a PE = 2 SV = 1 (P62835)	P62835	21 kDa	27.752	0
Cluster of Serine/threonine-protein phosphatase 2A 65 kDa regulatory subunit A alpha isoform OS = Mus musculus GN	Q76MZ3	65 kDa	18.501	0

Table S1. *Cont.*

Identified Proteins	Accession Number	Molecular Weight	Quantitative Value	
			1 h	24 h
Ras-related protein Rab-11A OS = Mus musculus GN = Rab11a PE = 3 SV = 1	E9Q3P9	17 kDa	37.002	11.441
Regenerating islet-derived protein 3-gamma OS = Mus musculus GN = Reg3g PE = 1 SV = 1	O09049	19 kDa	18.501	34.323
26S proteasome non-ATPase regulatory subunit 3 OS = Mus musculus GN = Psmd3 PE = 1 SV = 3	P14685	61 kDa	0	0
Cofilin-1 OS = Mus musculus GN = Cfl1 PE = 1 SV = 3	P18760	19 kDa	83.255	91.529
Cluster of Hydroxyacylglutathione hydrolase, mitochondrial (Fragment) OS = Mus musculus GN = Hagh PE = 1 SV = 1 (E9PY)	E9PYA3	26 kDa	18.501	22.882
Cluster of Dual-specificity protein phosphatase 3 OS = Mus musculus GN = Dusp3 PE = 4 SV = 1 (B1AQF4)	B1AQF4	23 kDa	83.255	11.441
Cluster of Vascular cell adhesion protein 1 OS = Mus musculus GN = Vcam1 PE = 1 SV = 1 (P29533)	P29533 [2]	81 kDa	18.501	0
Cluster of Thioredoxin domain-containing protein 5 OS = Mus musculus GN = Txndc5 PE = 1 SV = 2 (Q91W90)	Q91W90	46 kDa	0	0
Cluster of Actin-related protein 3 OS = Mus musculus GN = Actr3 PE = 1 SV = 3 (Q99JY9)	Q99JY9	47 kDa	0	57.206
Cluster of Obg-like ATPase 1 OS = Mus musculus GN = Ola1 PE = 1 SV = 1 (Q9CZ30)	Q9CZ30	45 kDa	0	0
Actin-related protein 2 OS = Mus musculus GN = Actr2 PE = 1 SV = 1	P61161	45 kDa	0	45.765
Dermatopontin OS = Mus musculus GN = Dpt PE = 2 SV = 1	Q9QZZ6	24 kDa	18.501	22.882
Ig lambda-1 chain C region OS = Mus musculus PE = 1 SV = 1	P01843	12 kDa	0.92506	11.441
SPARC OS = Mus musculus GN = Sparc PE = 1 SV = 1	P07214 (+1)	34 kDa	0	22.882
Ig heavy chain V region MOPC 21 (Fragment) OS = Mus musculus PE = 1 SV = 1	P01783	15 kDa	37.002	12.585
Destrin OS = Mus musculus GN = Dstn PE = 1 SV = 3	Q9R0P5	19 kDa	0.92506	45.765
Ferritin heavy chain OS = Mus musculus GN = Fth1 PE = 1 SV = 2	P09528	21 kDa	27.752	22.882
Acyl-coenzyme A thioesterase 13 OS = Mus musculus GN = Acot13 PE = 1 SV = 1	Q9CQR4	15 kDa	37.002	0
Ig kappa chain V-II region 7S34.1 OS = Mus musculus PE = 1 SV = 1	P01630	12 kDa	37.002	57.206
Cluster of Glycogenin-1 OS = Mus musculus GN = Gyg PE = 4 SV = 1 (K3W4S6)	K3W4S6 [2]	42 kDa	37.002	0
Cluster of 60S ribosomal protein L18a OS = Mus musculus GN = Rpl18a PE = 1 SV = 1 (P62717)	P62717	21 kDa	0	0
Ig kappa chain V-V region MOPC 41 OS = Mus musculus GN = Gm5571 PE = 1 SV = 1	P01639	14 kDa	37.002	34.323
Ig heavy chain V-III region A4 OS = Mus musculus PE = 1 SV = 1	P01796 (+2)	13 kDa	37.002	45.765
Isoform Smooth muscle of Myosin light polypeptide 6 OS = Mus musculus GN = Myl6	Q60605-2	17 kDa	0.92506	12.585
Endoplasmic reticulum resident protein 29 OS = Mus musculus GN = Erp29 PE = 1 SV = 2	P57759	29 kDa	18.501	22.882
Protein AI182371 OS = Mus musculus GN = AI182371 PE = 4 SV = 2	A2AS37 (+1)	40 kDa	18.501	34.323
Ras-related protein Rab-7a OS = Mus musculus GN = Rab7a PE = 1 SV = 2	P51150	23 kDa	27.752	34.323
Cystatin-C OS = Mus musculus GN = Cst3 PE = 2 SV = 2	P21460	16 kDa	18.501	22.882
Protein disulfide-isomerase A6 OS = Mus musculus GN = Pdia6 PE = 1 SV = 3	Q922R8	48 kDa	0	11.441
Cluster of F-actin-capping protein subunit alpha-2 OS = Mus musculus GN = Capza2 PE = 1 SV = 3 (P47754)	P47754	33 kDa	0.92506	22.882
Cluster of Ribonuclease 4 OS = Mus musculus GN = Rnase4 PE = 2 SV = 1 (Q9JJH1)	Q9JJH1	17 kDa	0.92506	45.765
Cluster of ADP/ATP translocase 1 OS = Mus musculus GN = Slc25a4 PE = 1 SV = 4 (P48962)	P48962	33 kDa	27.752	0
Cluster of Cathepsin D OS = Mus musculus GN = Ctsd PE = 1 SV = 1 (P18242)	P18242	45 kDa	0	0
Cluster of MCG130175, isoform CRA_b OS = Mus musculus GN = BC100530 PE = 2 SV = 1 (Q497J0)	Q497J0	11 kDa	0	80.088
Pentraxin-related protein PTX3 OS = Mus musculus GN = Ptx3 PE = 1 SV = 2	P48759	42 kDa	0	34.323

Table S1. *Cont.*

Identified Proteins	Accession Number	Molecular Weight	Quantitative Value	
			1 h	24 h
Coagulation factor XIII B chain OS = Mus musculus GN = F13b PE = 1 SV = 2	Q07968	76 kDa	0.92506	0
Glutathione S-transferase A4 OS = Mus musculus GN = Gsta4 PE = 1 SV = 3	P24472	26 kDa	0	22.882
Eukaryotic peptide chain release factor subunit 1 OS = Mus musculus GN = Etf1 PE = 1 SV = 4	Q8BWY3	49 kDa	27.752	0
Pyruvate carboxylase OS = Mus musculus GN = Pcx PE = 1 SV = 1	E9QPD7 (+2)	130 kDa	18.501	0
Pyruvate dehydrogenase E1 component subunit beta, mitochondrial OS = Mus musculus GN = Pdhb PE = 1 SV = 1	Q9D051	39 kDa	0	0
Cluster of Glucose-6-phosphate 1-dehydrogenase X OS = Mus musculus GN = G6pdx PE = 1 SV = 3 (Q00612)	Q00612	59 kDa	0	11.441
Cluster of Ig kappa chain V-VI region XRPC 44 OS = Mus musculus PE = 1 SV = 1 (P01675)	P01675 [4]	12 kDa	0.92506	0
Cluster of Rab GDP dissociation inhibitor alpha OS = Mus musculus GN = Gdi1 PE = 1 SV = 3 (P50396)	P50396	51 kDa	0	0
Annexin A1 OS = Mus musculus GN = Anxa1 PE = 1 SV = 2	P10107	39 kDa	0	57.206
Ribose-5-phosphate isomerase OS = Mus musculus GN = Rpia PE = 2 SV = 2	P47968	32 kDa	18.501	34.323
40S ribosomal protein S13 OS = Mus musculus GN = Rps13 PE = 1 SV = 2	P62301	17 kDa	0	0
Peptidyl-prolyl cis-trans isomerase FKBP3 OS = Mus musculus GN = Fkbp3 PE = 1 SV = 2	Q62446	25 kDa	18.501	11.441
Serpin B6 OS = Mus musculus GN = Serpinb6a PE = 1 SV = 1	F8WIV2	45 kDa	0.92506	22.882
Ubiquitin carboxyl-terminal hydrolase isozyme L3 OS = Mus musculus GN = Uchl3 PE = 1 SV = 2	Q9JKB1	26 kDa	18.501	11.441
Ras-related C3 botulinum toxin substrate 2 OS = Mus musculus GN = Rac2 PE = 2 SV = 1	Q05144	21 kDa	27.752	80.088
Carboxypeptidase Q OS = Mus musculus GN = Cpq PE = 2 SV = 1	Q9WVJ3 (+1)	52 kDa	0.92506	0
60S ribosomal protein L23 OS = Mus musculus GN = Rpl23 PE = 1 SV = 1	P62830	15 kDa	0	11.441
Isoleucine--tRNA ligase, cytoplasmic OS = Mus musculus GN = Iars PE = 2 SV = 2	Q8BU30	144 kDa	18.501	0
Cluster of Carbonyl reductase [NADPH] 2 OS = Mus musculus GN = Cbr2 PE = 1 SV = 1 (P08074)	P08074	26 kDa	18.501	22.882
Cluster of Mannosyl-oligosaccharide 1,2-alpha-mannosidase IA OS = Mus musculus GN = Man1a1 PE = 1 SV = 1 (P45700)	P45700	73 kDa	0.92506	0
Glyceraldehyde-3-phosphate dehydrogenase, testis-specific OS = Mus musculus GN = Gapdhs PE = 2 SV = 1	Q64467 (+1)	48 kDa	24.977	22.882
Cluster of Serine/threonine-protein phosphatase 2A 65 kDa regulatory subunit A beta isoform OS = Mus musculus GN =	H3BLE7	66 kDa	27.752	0
Isoform Short of Heterogeneous nuclear ribonucleoprotein A1 OS = Mus musculus GN = Hnrnpa1	P49312-2	29 kDa	0.92506	0
40S ribosomal protein S14 OS = Mus musculus GN = Rps14 PE = 2 SV = 3	P62264	16 kDa	0	45.765
Carbonyl reductase [NADPH] 3 OS = Mus musculus GN = Cbr3 PE = 2 SV = 1	Q8K354	31 kDa	0.92506	34.323
Pro-cathepsin H OS = Mus musculus GN = Ctsh PE = 2 SV = 2	P49935	37 kDa	0	22.882
Platelet-activating factor acetylhydrolase IB subunit beta OS = Mus musculus GN = Pafah1b2 PE = 1 SV = 2	Q61206	26 kDa	18.501	34.323
Biliverdin reductase A OS = Mus musculus GN = Blvra PE = 2 SV = 1	Q9CY64	34 kDa	0	0
Eosinophil cationic-type ribonuclease 3 OS = Mus musculus GN = Ear3 PE = 3 SV = 1	O35290	18 kDa	0.92506	11.441
Heterogeneous nuclear ribonucleoproteins A2/B1 OS = Mus musculus GN = Hnrnpa2b1 PE = 1 SV = 2	O88569	37 kDa	0	34.323
Programmed cell death 6-interacting protein OS = Mus musculus GN = Pdcd6ip PE = 1 SV = 3	Q9WU78	96 kDa	0.92506	0
Ubiquitin-conjugating enzyme E2 K OS = Mus musculus GN = Ube2k PE = 1 SV = 3	P61087	22 kDa	37.002	11.441
Ubiquitin thioesterase OTUB1 OS = Mus musculus GN = Otub1 PE = 1 SV = 2	Q7TQI3	31 kDa	18.501	0
N(G),N(G)-dimethylarginine dimethylaminohydrolase 2 OS = Mus musculus GN = Ddah2 PE = 1 SV = 1	Q99LD8	30 kDa	18.501	11.441
Actin-related protein 2/3 complex subunit 5 OS = Mus musculus GN = Arpc5 PE = 2 SV = 3	Q9CPW4	16 kDa	18.501	22.882

Table S1. *Cont.*

Identified Proteins	Accession Number	Molecular Weight	Quantitative Value	
			1 h	24 h
Cadherin-1 OS = Mus musculus GN = Cdh1 PE = 1 SV = 1	P09803	98 kDa	0	0
Cluster of Ig kappa chain V-III region PC 7175 OS = Mus musculus PE = 1 SV = 1 (P01671)	P01671	12 kDa	46.253	14.873
Thyroxine-binding globulin OS = Mus musculus GN = Serpina7 PE = 2 SV = 1	P61939	47 kDa	0	34.323
Eukaryotic initiation factor 4A-II OS = Mus musculus GN = Eif4a2 PE = 3 SV = 1	E9Q561	36 kDa	27.752	0
Cluster of Retinal dehydrogenase 1 OS = Mus musculus GN = Aldh1a1 PE = 1 SV = 5 (P24549)	P24549	54 kDa	0	0
Isoform 2 of Guanidinoacetate N-methyltransferase OS = Mus musculus GN = Gamt	O35969-2	28 kDa	37.002	0
Dual specificity phosphatase DUPD1 OS = Mus musculus GN = Dupd1 PE = 2 SV = 1	Q8BK84	24 kDa	27.752	0
EMILIN-1 OS = Mus musculus GN = Emilin1 PE = 1 SV = 1	Q99K41	108 kDa	18.501	0
Cluster of Isoform 2 of Alpha-synuclein OS = Mus musculus GN = Snca (O55042-2)	O55042-2	12 kDa	18.501	34.323
Myosin light chain 3 OS = Mus musculus GN = Myl3 PE = 1 SV = 4	P09542	22 kDa	0	14.873
60S ribosomal protein L30 OS = Mus musculus GN = Rpl30 PE = 2 SV = 2	P62889	13 kDa	0.92506	45.765
60S acidic ribosomal protein P2 OS = Mus musculus GN = Rplp2 PE = 1 SV = 3	P99027	12 kDa	0.92506	22.882
Ubiquitin carboxyl-terminal hydrolase OS = Mus musculus GN = Usp5 PE = 1 SV = 1	Q3U4W8	93 kDa	0	0
Phospholipid transfer protein OS = Mus musculus GN = Pltp PE = 4 SV = 1	A2A5K2	49 kDa	0.92506	0
Tubulin beta-1 chain OS = Mus musculus GN = Tubb1 PE = 1 SV = 1	A2AQ07	50 kDa	18.501	11.441
Cathelin-related antimicrobial peptide OS = Mus musculus GN = Camp PE = 2 SV = 1	P51437	20 kDa	0	45.765
Heterogeneous nuclear ribonucleoprotein A/B OS = Mus musculus GN = Hnrnpab PE = 1 SV = 1	Q20BD0 (+2)	36 kDa	0	34.323
AP-2 complex subunit alpha-2 OS = Mus musculus GN = Ap2a2 PE = 1 SV = 2	P17427	104 kDa	0.92506	0
Apolipoprotein C-IV OS = Mus musculus GN = Apoc4 PE = 2 SV = 1	Q61268	14 kDa	37.002	34.323
Epididymal secretory protein E1 OS = Mus musculus GN = Npc2 PE = 1 SV = 1	Q9Z0J0	16 kDa	0	22.882
Cluster of EF-hand domain-containing protein D2 OS = Mus musculus GN = Efhd2 PE = 1 SV = 1 (Q8C845)	Q8C845	27 kDa	18.501	0
Cluster of Coronin-1A OS = Mus musculus GN = Coro1a PE = 1 SV = 5 (O89053)	O89053	51 kDa	0	22.882
Heat shock protein beta-2 OS = Mus musculus GN = Hspb2 PE = 2 SV = 2	Q99PR8	20 kDa	27.752	0
Hypoxanthine-guanine phosphoribosyltransferase OS = Mus musculus GN = Hprt1 PE = 1 SV = 3	P00493	25 kDa	0	11.441
Ras-related protein Rab-18 OS = Mus musculus GN = Rab18 PE = 2 SV = 2	P35293	23 kDa	27.752	0
Acylpyruvase FAHD1, mitochondrial OS = Mus musculus GN = Fahd1 PE = 1 SV = 2	Q8R0F8	25 kDa	0	11.441
Cluster of COP9 signalosome complex subunit 7a (Fragment) OS = Mus musculus GN = Cops7a PE = 4 SV = 2 (D3Z440)	D3Z440 [2]	25 kDa	18.501	0
Rho-related GTP-binding protein RhoB OS = Mus musculus GN = Rhob PE = 1 SV = 1	P62746	22 kDa	0.92506	0
Cluster of D-dopachrome decarboxylase OS = Mus musculus GN = Ddt PE = 1 SV = 3 (O35215)	O35215	13 kDa	27.752	11.441
Cluster of ATP synthase subunit O, mitochondrial OS = Mus musculus GN = Atp5o PE = 1 SV = 1 (Q9DB20)	Q9DB20	23 kDa	18.501	0
Cluster of Ras-related protein Rab-2A OS = Mus musculus GN = Rab2a PE = 1 SV = 1 (P53994)	P53994	24 kDa	18.501	0
Cluster of L-selectin OS = Mus musculus GN = Sell PE = 2 SV = 1 (P18337)	P18337	42 kDa	18.501	0
COP9 signalosome complex subunit 8 OS = Mus musculus GN = Cops8 PE = 1 SV = 1	Q8VBV7	23 kDa	27.752	0
Apoptosis-associated speck-like protein containing a CARD OS = Mus musculus GN = Pycard PE = 1 SV = 1	Q9EPB4	21 kDa	0	22.882
Protein MEMO1 OS = Mus musculus GN = Memo1 PE = 1 SV = 1	Q91VH6	34 kDa	0	0

Table S1. *Cont.*

Identified Proteins	Accession Number	Molecular Weight	Quantitative Value	
			**1 h**	**24 h**
Serine/threonine-protein phosphatase 2A catalytic subunit alpha isoform OS = Mus musculus GN = Ppp2ca PE = 1 SV = 1	P63330	36 kDa	18.501	0
Histone H2A OS = Mus musculus GN = H2afz PE = 2 SV = 1	Q3UA95	11 kDa	27.752	16.018
L-lactate dehydrogenase C chain OS = Mus musculus GN = Ldhc PE = 1 SV = 2	P00342	36 kDa	25.902	80.088
Cluster of Elongation factor 1-beta OS = Mus musculus GN = Eef1b PE = 1 SV = 5 (O70251)	O70251	25 kDa	18.501	22.882
Cluster of Isopentenyl-diphosphate Delta-isomerase 1 OS = Mus musculus GN = Idi1 PE = 2 SV = 1 (P58044)	P58044	26 kDa	0	0
Cluster of Malectin OS = Mus musculus GN = Mlec PE = 2 SV = 2 (Q6ZQI3)	Q6ZQI3	32 kDa	0	0
Alpha-soluble NSF attachment protein OS = Mus musculus GN = Napa PE = 1 SV = 1	Q9DB05	33 kDa	27.752	0
2,4-dienoyl-CoA reductase, mitochondrial OS = Mus musculus GN = Decr1 PE = 1 SV = 1	Q9CQ62	36 kDa	18.501	0
40S ribosomal protein S8 OS = Mus musculus GN = Rps8 PE = 1 SV = 2	P62242	24 kDa	0	22.882
Cluster of Ribose-phosphate pyrophosphokinase 1 OS = Mus musculus GN = Prps1 PE = 1 SV = 4 (Q9D7G0)	Q9D7G0	35 kDa	0	22.882
Ketimine reductase mu-crystallin OS = Mus musculus GN = Crym PE = 1 SV = 1	O54983	34 kDa	0	0
NAD(P)H-hydrate epimerase OS = Mus musculus GN = Apoa1bp PE = 1 SV = 1	Q8K4Z3	31 kDa	18.501	0
Biglycan OS = Mus musculus GN = Bgn PE = 2 SV = 1	P28653	42 kDa	0	22.882
SH3 domain-binding glutamic acid-rich-like protein OS = Mus musculus GN = Sh3bgrl PE = 3 SV = 1	Q9JJU8	13 kDa	0	11.441
Vesicle-associated membrane protein-associated protein A OS = Mus musculus GN = Vapa PE = 1 SV = 2	Q9WV55	28 kDa	0.92506	0
Cluster of Ig kappa chain V-III region PC 7043 OS = Mus musculus PE = 1 SV = 1 (P01665)	P01665 [2]	12 kDa	27.752	13.729
Cluster of Capping protein (Actin filament), gelsolin-like OS = Mus musculus GN = Capg PE = 1 SV = 1 (Q99LB4)	Q99LB4	39 kDa	0	11.441
Cluster of Aldehyde dehydrogenase, mitochondrial OS = Mus musculus GN = Aldh2 PE = 1 SV = 1 (P47738)	P47738	57 kDa	0	0
Plasminogen activator inhibitor 2, macrophage OS = Mus musculus GN = Serpinb2 PE = 2 SV = 1	P12388	46 kDa	0	22.882
Transcription elongation factor B polypeptide 1 OS = Mus musculus GN = Tceb1 PE = 1 SV = 1	P83940	12 kDa	18.501	0
Ig kappa chain V-I region S107A OS = Mus musculus GN = Igkv7-33 PE = 4 SV = 1	P01632	13 kDa	55.503	22.882
Vesicle-associated membrane protein, associated protein B and C OS = Mus musculus GN = Vapb PE = 2 SV = 1	Q8BH80 (+1)	27 kDa	18.501	0
Guanine nucleotide-binding protein G(I)/G(S)/G(T) subunit beta-3 OS = Mus musculus GN = Gnb3 PE = 1 SV = 2	Q61011	37 kDa	0	0
Serum amyloid A-1 protein OS = Mus musculus GN = Saa1 PE = 1 SV = 2	P05366	14 kDa	0	16.018
60S acidic ribosomal protein P1 OS = Mus musculus GN = Rplp1 PE = 2 SV = 1	P47955	11 kDa	0	22.882
GTP-binding protein SAR1b OS = Mus musculus GN = Sar1b PE = 1 SV = 1	Q9CQC9	22 kDa	46.253	0
Eukaryotic translation initiation factor 3 subunit L OS = Mus musculus GN = Eif3l PE = 1 SV = 1	Q8QZY1	67 kDa	0	0
Serine/threonine-protein phosphatase 2A 55 kDa regulatory subunit B alpha isoform OS = Mus musculus GN = Ppp2r2a	Q6P1F6	52 kDa	0.92506	0
ATP-dependent 6-phosphofructokinase, liver type OS = Mus musculus GN = Pfkl PE = 1 SV = 4	P12382	85 kDa	0	0
Myosin light chain 4 OS = Mus musculus GN = Myl4 PE = 2 SV = 3	P09541	21 kDa	0	0
Ig heavy chain V region 3 OS = Mus musculus GN = Ighv1-61 PE = 1 SV = 1	P01749	13 kDa	27.752	11.441
Cluster of Peroxiredoxin-4 (Fragment) OS = Mus musculus GN = Prdx4 PE = 4 SV = 1 (B1AZS9)	B1AZS9	26 kDa	64.754	45.765
Cluster of Prefoldin subunit 2 OS = Mus musculus GN = Pfdn2 PE = 2 SV = 2 (O70591)	O70591	17 kDa	18.501	0
F-actin-capping protein subunit alpha-1 OS = Mus musculus GN = Capza1 PE = 1 SV = 4	P47753 (+1)	33 kDa	0	11.441
Ig kappa chain V-III region PC 3741/TEPC 111 OS = Mus musculus PE = 1 SV = 1	P01660	12 kDa	46.253	10.297

Table S1. *Cont.*

Identified Proteins	Accession Number	Molecular Weight	Quantitative Value	
			1 h	24 h
PRA1 family protein 3 OS = Mus musculus GN = Arl6ip5 PE = 1 SV = 2	Q8R5J9	22 kDa	0	0
GTP-binding protein SAR1a OS = Mus musculus GN = Sar1a PE = 2 SV = 1	Q99JZ4	22 kDa	37.002	11.441
Ribonuclease UK114 OS = Mus musculus GN = Hrsp12 PE = 1 SV = 3	P52760	14 kDa	0.92506	0
Ig kappa chain V-III region ABPC 22/PC 9245 OS = Mus musculus PE = 1 SV = 1	P01662 (+1)	12 kDa	18.501	68.647
Cluster of Glia maturation factor gamma OS = Mus musculus GN = Gmfg PE = 1 SV = 1 (Q9ERL7)	Q9ERL7	17 kDa	0	34.323
Tubulin polymerization-promoting protein family member 3 OS = Mus musculus GN = Tppp3 PE = 1 SV = 1	Q9CRB6	19 kDa	0.92506	22.882
Cluster of Heme oxygenase 1 OS = Mus musculus GN = Hmox1 PE = 1 SV = 1 (P14901)	P14901	33 kDa	0	22.882
Diphosphoinositol polyphosphate phosphohydrolase 1 OS = Mus musculus GN = Nudt3 PE = 3 SV = 1	H3BLR8 (+1)	14 kDa	18.501	0
H-2 class I histocompatibility antigen, D-K alpha chain OS = Mus musculus GN = H2-D1 PE = 1 SV = 1	P14426	41 kDa	18.501	11.441
Heterogeneous nuclear ribonucleoprotein D0 (Fragment) OS = Mus musculus GN = Hnrnpd PE = 1 SV = 1	E9Q5B6	12 kDa	0	11.441
Astrocytic phosphoprotein PEA-15 OS = Mus musculus GN = Pea15 PE = 1 SV = 1	Q62048 (+1)	15 kDa	0	22.882
Calmodulin-4 OS = Mus musculus GN = Calm4 PE = 2 SV = 2	Q9JM83	17 kDa	0	22.882
Protein FAM49B OS = Mus musculus GN = Fam49b PE = 2 SV = 1	Q921M7	37 kDa	18.501	11.441
Ig lambda-1 chain V region OS = Mus musculus PE = 1 SV = 2	P01723 (+1)	12 kDa	18.501	0
